# Persistent COVID-19 symptoms in a community study of 606,434 people in England

**DOI:** 10.1038/s41467-022-29521-z

**Published:** 2022-04-12

**Authors:** Matthew Whitaker, Joshua Elliott, Marc Chadeau-Hyam, Steven Riley, Ara Darzi, Graham Cooke, Helen Ward, Paul Elliott

**Affiliations:** 1 School of Public Health, Imperial College London, London, UK; 2 MRC Centre for Environment and Health, Imperial College London, London, UK; 3 Imperial College Healthcare NHS Trust, London, UK; 4 Department of Infectious Disease, Imperial College London, London, UK; 5 MRC Centre for Global Infectious Disease Analysis, Imperial College London, London, UK; 6 Abdul Latif Jameel Institute for Disease & Emergency Analytics, Imperial College London, London, UK; 7 Institute of Global Health Innovation at Imperial College London, London, UK; 8 National Institute for Health Research Imperial Biomedical Research Centre, London, UK; 9 Health Data Research (HDR) UK London at Imperial College, London, UK; 10 UK Dementia Research Institute at Imperial College, London, UK

**Keywords:** Viral infection, Data mining, SARS-CoV-2, Epidemiology

## Abstract

Long COVID remains a broadly defined syndrome, with estimates of prevalence and duration varying widely. We use data from rounds 3–5 of the REACT-2 study (*n* **=** 508,707; September 2020 – February 2021), a representative community survey of adults in England, and replication data from round 6 (*n* **=** 97,717; May 2021) to estimate the prevalence and identify predictors of persistent symptoms lasting 12 weeks or more; and unsupervised learning to cluster individuals by reported symptoms. At 12 weeks in rounds 3–5, 37.7% experienced at least one symptom, falling to 21.6% in round 6. Female sex, increasing age, obesity, smoking, vaping, hospitalisation with COVID-19, deprivation, and being a healthcare worker are associated with higher probability of persistent symptoms in rounds 3–5, and Asian ethnicity with lower probability. Clustering analysis identifies a subset of participants with predominantly respiratory symptoms. Managing the long-term sequelae of COVID-19 will remain a major challenge for affected individuals and their families and for health services.

## Abstract (web summary)

This study characterises Long COVID using data from the REACT-2 community-based study in England. It estimates that 38% (in autumn/winter 2020/21) and 22% (in spring 2021) of people reported at least one symptom 12 weeks after symptom onset; identifies risk factors for persistent symptoms; and finds evidence of symptom clustering.

## Introduction

The UK has experienced one of the largest epidemics of COVID-19 in Europe. As a new disease, the natural history beyond the immediate illness and the possible long-term sequelae remain largely unknown. As well as the acute risk of hospitalisation and death from COVID-19, some people who develop symptoms have a prolonged and debilitating illness that may continue for weeks or months^1–5^. This has been called post-COVID syndrome^6^ or Long COVID, a term first coined by people sharing their experience of ongoing symptoms on social media and establishing support groups^7^.

The frequency, nature and duration of persistent symptoms from COVID-19 are poorly understood and represent a major knowledge gap if effective treatments and management strategies are to be developed. Reported symptoms include severe fatigue, breathlessness, chest pain or heaviness, fever, palpitations, cognitive impairment (‘brain fog’), loss of sense of smell (anosmia), loss of sense of taste (ageusia), skin rash and joint pain or swelling^1–5^. Estimates of symptom prevalence and persistence vary substantially, arguably due to heterogeneous study designs and syndrome definitions^8–11^. It has been suggested that Long COVID describes a group of disparate conditions, including post-viral syndromes, long-term tissue or organ damage and ongoing inflammation^3,9,12,13^.

Occurrence of Long COVID appears to be associated with the severity of COVID-19; for example, high prevalence of persistent symptoms has been reported among people hospitalised with COVID-19^14–16^. The number of acute symptoms has also been associated with risk of Long COVID, alongside older age and female sex^8^.

While many Long COVID studies so far have focused on hospitalised COVID-19 cases^14–19^, here we report data from random community-based samples of the population in England. These involved more than 600,000 people who took part in rounds three to five (main analysis) and round six (replication) of the Real-Time Assessment of Community Transmission-2 (REACT-2) study between September 2020 and May 2021. Among participants reporting symptoms lasting 12 weeks or more following suspected or confirmed COVID-19, we estimate symptom prevalence, investigate co-occurrence of symptoms and assess risk factors for persistence of symptoms.

## Results

**Fig. 1:**
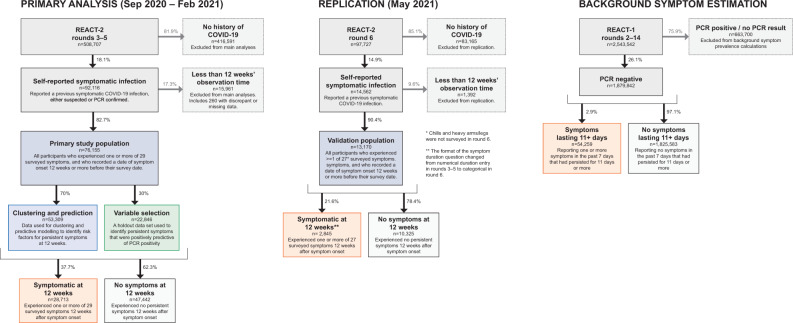
Study population flow chart. An overview of primary and replication study population size, with exclusions and proportion of participants experiencing symptoms 12 weeks after symptom onset.

Figure 1 shows the study design and population. A total of 508,707 people took part in REACT-2 rounds 3–5, and 97,727 in REACT-2 round 6 (excluding a ‘booster’ sample of additional people recruited at ages 55 years and over), with response rates of 29.4% and 29.9% respectively. Compared to responders, non-responders were more likely to be men, younger (18–24 years) or older (>75 years) adults and live in more deprived areas (Supplementary Table 1).

A total of 92,116 respondents reported previous COVID-19 in rounds 3–5, and 14,562 in round 6, giving a weighted prevalence of 19.2% (19.1,19.3) and 17.9% (17.7,18.0) respectively.

### Prevalence of persistent symptoms

Table 1 shows the proportion of people with COVID-19 who still reported one or more, or three or more, of 29 symptoms at 12 weeks after symptom onset. At 12 weeks, 37.7% (37.4,38.1) of those in rounds 3–5 reported one or more symptoms, and 17.5% (17.2,17.7) reported three or more; in round 6, these figures were 21.6% (20.9,22.3) and 11.9% (11.4,12.5), respectively. For rounds 3–5, these translated to a weighted population prevalence of 5.80% (5.73,5.86) for having, or having had, one or more persistent symptoms for 12 weeks or more, and 2.23% (2.19,2.27) for three or more persistent symptoms. In round 6 the equivalent percentages were 3.06% (2.98,3.14) and 1.61 (1.56,1.67), respectively, for 27 symptoms in common with rounds 3–5 (Supplementary Table 5), increasing to 3.26% (3.18,3.34) and 1.86% (1.80,1.92) for one and three symptoms respectively if all 35 symptoms surveyed in round 6 are included (Supplementary Table 6).

**Table 1:** Proportions of respondents in (i) rounds 3–5 and (ii) round 6 who still reported one or more (or three or more) symptoms 12 weeks after initial symptom onset.

	*n*	*n* with prior symptomatic COVID-19 and 12 weeks’ observation time	% with one or more symptoms at 12 weeks	% with three or more symptoms at 12 weeks
Rounds 3–5	508,707	76,155	37.7 [37.4–38.1]	17.47 [17.2–17.7]
Round 6	97,727	13,170	21.6 [20.9–22.3]	11.94 [11.4–12.5]
Round 6 (extended symptom list)			22.8 [22.1–23.5]	13.82 [13.2–14.4]

**Fig. 2:**
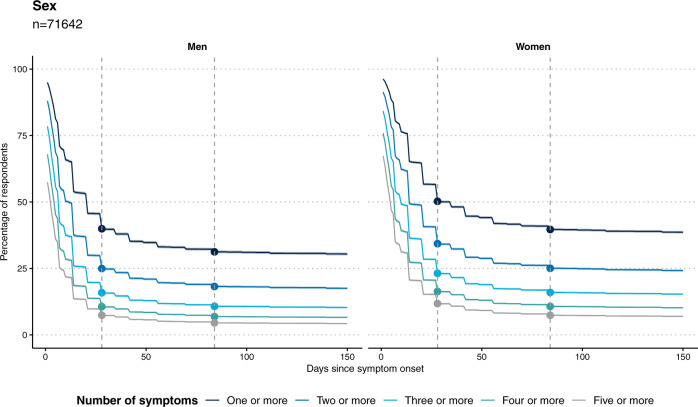
Persistence of symptoms over time. Plots showing persistence of symptoms as a proportion of those who reported symptoms at any time, among *n* = 71,642 respondents for whom we had 150 days’ observation time. Women have higher rates of persistent symptoms; a slower decline in symptom prevalence is observed after 12 weeks in both sexes. The vertical dashed lines show 4 and 12 weeks post symptom onset, respectively.

Figure 2 shows the proportion of people with one or multiple symptoms over time since symptom onset. There was a rapid drop-off in symptom reporting by 4 weeks, a further, smaller drop by 12 weeks, but then limited further decline up to ~22 weeks for both men and women, with higher prevalence of symptoms at each time point among women.

**Fig. 3:**
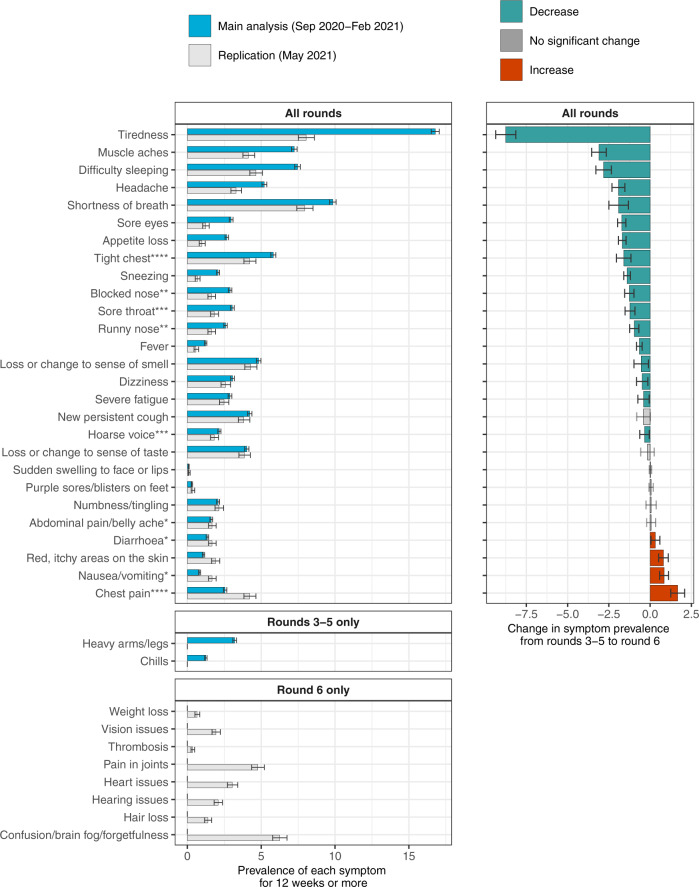
Symptom prevalence in September 2020–February 2021, and in May 2021. Prevalence of 37 symptoms surveyed across rounds 3–6 of REACT-2. Top panel shows symptoms that were surveyed in all rounds (*n* = 606,434 observations); middle panel shows symptoms surveyed in rounds 3–5 only (*n* = 508,707 observations); bottom panel shows symptoms surveyed in round 6 only (*n* = 97,727 observations). Right panel compares symptom prevalence in the main study cohort (REACT-2 rounds 3–5, surveyed between October 2020 and February 2021) with the replication cohort (REACT-2 round 6, surveyed in May 2021). Asterisks indicate symptoms that were grouped in the round 6 survey. Green bars in the right panel indicate a decrease in symptom prevalence in round 6 compared with rounds 3–5. Error bars indicate 95% binomial confidence intervals of the prevalence.

In rounds 3–5, the most prevalent persistent symptom was tiredness at 16.8% (16.5,17.1), whereas in round 6 reporting of tiredness was much lower at 8.0% (7.5,8.6) (Fig. 3, Supplementary Table 2). Smaller declines in prevalence from rounds 3–5 to round 6 were observed for 16 of the other 26 symptoms that were common to all four rounds, while increases were observed for four symptoms (Fig. 3).

### Risk factors for persistent symptoms at 12 weeks

**Fig. 4:**
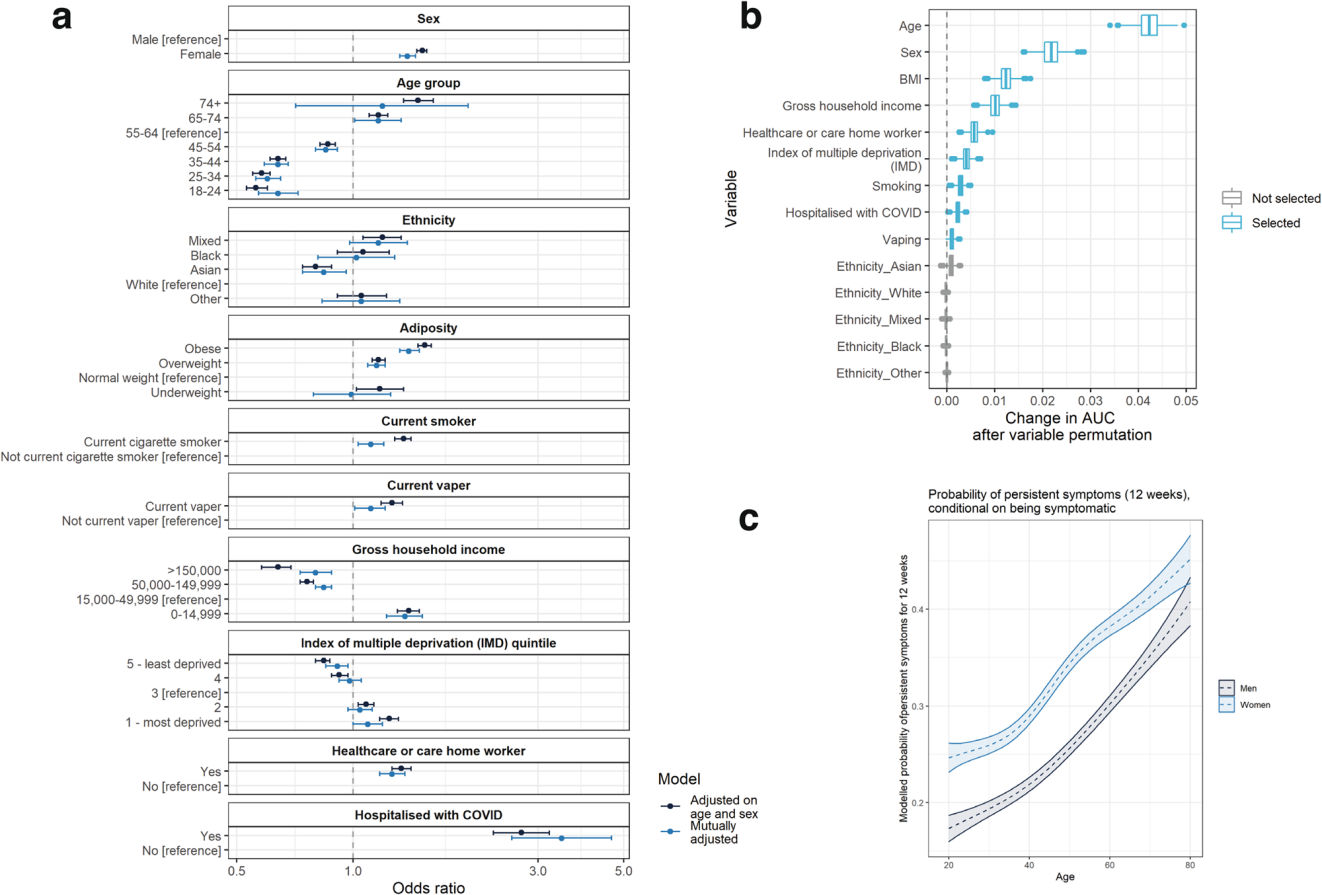
Modelling of persistent symptoms as a function of biological and demographic variables. **a** Logistic regression models with one or more symptoms at 12 weeks (y/n) as the binary outcome variable, both adjusted for age-sex and mutually adjusted*; **b** mean contribution to area under the curve (AUC) that each variable makes to a multivariable boosted tree model, derived by permuting each variable in turn (1000× to obtain a distribution) and quantifying the change in model performance; **c** modelled probability of persistent symptoms at 12 weeks as a function of age and sex, using generalised additive models with splines on age and interactions between age and sex. All models were fit on *n* = 71,642 respondents for whom we had 150 days’ observation time. Age, sex, adiposity household income, healthcare/care home worker, deprivation, current smoker status and prior hospitalisation with COVID-19 are the strongest predictors of persistent symptoms in multivariable modelling, while Asian ethnicity is associated with a lower risk of persistent symptoms at 12 weeks. Box plots in panel **b** show median, first and third quartiles; whiskers indicate 1.5 × the interquartile range; data beyond this range are plotted as points. Note: Owing to missing data in some variables, the total *n* for the mutually adjusted model in panel **a** is 55,730.

Prevalence by sociodemographic and lifestyle factors is shown in Supplementary Tables 3–8. To test the independent effects of these factors on risk of persistent symptoms, we carried out age- and sex- and mutually adjusted logistic regression as well as multivariable analysis for variable selection. In rounds 3–5, the persistence of one or more symptoms for 12 weeks or more was associated with female sex, increasing age, being overweight or obese, smoking, vaping, hospitalisation with COVID-19, deprivation, low household income, and healthcare or care home workers, with odds ratios ranging from 1.38 (1.32,1.45) for female sex to 3.45 (2.57,4.64) for hospitalisation with COVID-19 (Fig. 4, Supplementary Table 9). Asian ethnicity was associated with lower risk of persistent symptoms compared to people of white ethnicity (OR: 0.84 [0.74,0.96]). In multivariable analysis for variable selection and ranking, the strongest predictors of persistent symptoms, in order, were age, sex, body mass index (BMI), household income, healthcare/care home worker, deprivation, smoking status, prior hospitalisation with COVID-19 and vaping status.

In generalised additive models (GAMs) with likelihood of symptom persistence at 12 weeks or more modelled as a smoothed function of sex and age, risk of persistent symptoms increased linearly with age in both men and women with an additional 3.5 percentage points of risk per decade of life. Women had ~8 percentage points higher risk than men at all ages (Fig. 4, Supplementary Fig. 1).

The results of the logistic modelling and variable selection in the replication data set (REACT-2 round 6, from May 2021), were similar (Supplementary Fig. 2), except that smoking, vaping and deprivation were not associated with persistent symptoms in multiple logistic regression, and in multivariable variable selection analysis, healthcare/care home worker status, gross household income and deprivation were not selected, while Asian ethnicity was. Statistical power for these analyses was lower, however, given the smaller sample size in round 6 compared with rounds 3–5.

### Clustering analysis

**Fig. 5:**
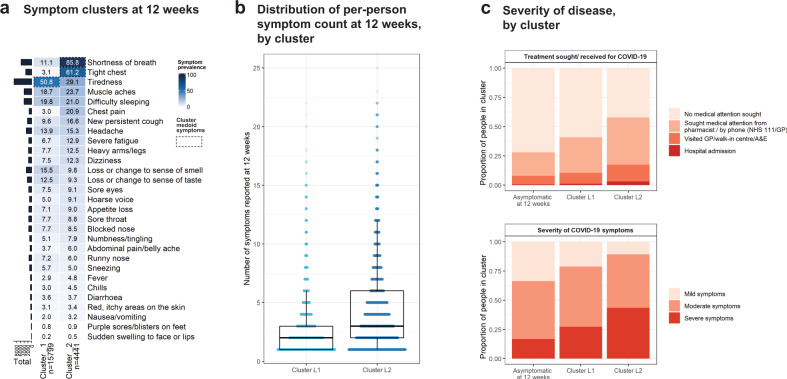
Results of clustering on symptom profile at 12 weeks. Clustering was conducted using CLARA (partitioning around medoids) algorithm. Two stable clusters were identified at 12 weeks. Cluster L1 (“tiredness cluster”) had high prevalence of tiredness. Cluster L2 (“respiratory cluster”) was a smaller subset of 4,441 participants who had high prevalence of shortness of breath and tight chest as well as chest pain. Panel **a** shows symptom prevalence by cluster. Panel **b** shows the distribution of symptom counts by cluster (median 2 symptoms for cluster L1 [*n* = 15,799] and 3 symptoms for cluster L2 [*n* = 4441]). Box plots in panel **b** show median, first and third quartiles; whiskers indicate 1.5*the interquartile range; data beyond this range are plotted as points. Panel **c** shows the self-reported symptom severity and medical treatment sought by cluster (with those who were no longer symptomatic at 12 weeks for comparison).

**Fig. 6:**
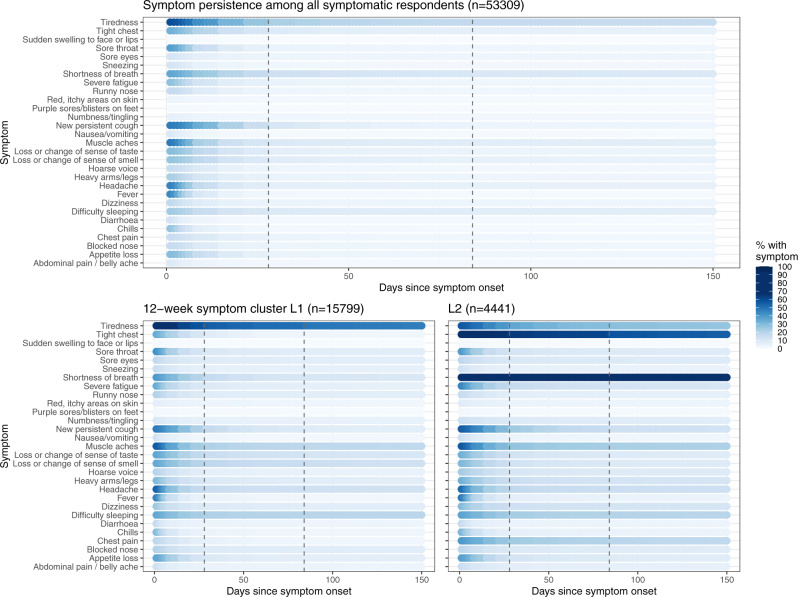
Persistence of individual symptoms, by symptom cluster. Persistence of symptoms for all symptomatic participants (top panel) and in 12-week symptoms clusters L1 and L2 (bottom panels). Dashed lines show 4 and 12 weeks post symptom onset, respectively.

In clustering analysis of the 20,240 participants in rounds 3–5 who were still symptomatic 12 weeks after initial symptom onset, two stable clusters of participants were identified based on symptom profiles at 12 weeks (Fig. 5, Supplementary Fig. 3). In bootstrap stability analysis, the clusters were recovered in 100% of stability bootstraps. There was high prevalence of persistent tiredness in Cluster L1 (*n* = 15,799), which co-occurred with muscle aches, difficulty sleeping and shortness of breath (Supplementary Fig. 4). Cluster L2 (*n* = 4441) had high prevalence of respiratory symptoms including shortness of breath and tight chest, as well as chest pain (Figs. 5,  6, Supplementary Fig. 4). The cluster medoids—the representative observations at the centre of each cluster—were a participant with only tiredness at 12 weeks (Cluster L1) and a participant with shortness of breath and tight chest at 12 weeks (Cluster L2) (Fig. 5, Supplementary Fig. 5). A higher proportion of people in the respiratory cluster (Cluster L2) reported severe symptoms at the time of their COVID-19 illness at 43.5% (42.0,44.9) than in Cluster L1 at 27.4% (26.7,28.1). Rates of hospitalisation were nearly three times as high in Cluster L2 (2.9% [2.5–3.5]) as in Cluster L1 (1.1% [0.9,1.3]) (Fig. 5, Supplementary Table 10).

In the replication data from round 6, clustering analysis again identified a subset of respondents (Cluster _R6_L_2; *n* = 1582) with high prevalence of shortness of breath, co-occurring with tight chest/chest pain, but also with high prevalence of tiredness, while another cluster (Cluster _R6_L_1; *n* = 1263) had high prevalence of loss of sense of smell and taste (Supplementary Fig. 6).

### Sensitivity analyses

In sensitivity analyses (rounds 3–5), we assessed the impact of (i) use of a reduced set of 15 symptoms associated with self-reported PCR-positivity (Supplementary Fig. 7), and (ii) restricting the study population to those who self-reported previous COVID-19 and tested positive on a lateral flow immunoassay (LFIA). Using 15 instead of 29 symptoms reduced the prevalence of persistent symptoms at 12 weeks by only four percentage points to 32.9% (32.6,33.2) and identified the same set of risk factors (Supplementary Fig. 8), while in the LFIA-positive subgroup the prevalence of persistent symptoms at 12 weeks was increased at 42.4% (41.6,43.2) (Supplementary Tables 3, 4, 11).

In clustering sensitivity analyses on round 3–5 data, the additional clustering methods (PAM using Dice distance and Jaccard distance) identified 5 and 6 clusters, respectively. In each case, two clusters with primarily respiratory symptoms were identified, which contained almost all the observations from the ‘respiratory’ cluster L2 in the main analysis (Supplementary Fig. 9). Across all methods and possible numbers of clusters, silhouette width was maximised when using two clusters with Hamming distance (presented in main analysis).

Latent class analysis identified one smaller class characterised by respiratory symptoms and higher overall symptom prevalence, and one larger class characterised predominantly by tiredness (Supplementary Fig. 10).

### Background symptom prevalence

We used REACT-1 data to estimate the background level of symptom reporting in PCR-negative adults (Methods). Among 1,879,842 PCR-negative adults during REACT-1 rounds 2–14 (June 2020–September 2021), average weighted prevalence of any of 26 symptoms lasting 11 or more days was 3.06% (3.04,3.09) (Supplementary Fig. 11).

We also used REACT-1 data to investigate potential differential recall bias by age. The proportion of PCR-positive individuals who reported any symptoms at time of infection was lower in the older age groups, consistent with the REACT-2 findings (Supplementary Table 12).

## Discussion

In this large community-based study of symptoms following COVID-19 among adults aged 18 years and above in England, participants reported high prevalence of persistent symptoms lasting 12 weeks or more. Estimates ranged from 5.8% of the adult population experiencing, or having experienced, one or more persistent symptoms post-COVID-19 (corresponding to over 2 million adults in England), to 2.2% for three or more persistent symptoms (just under a million adults in England) in rounds 3–5, and 3.1% and 1.6% for one and three persistent symptoms respectively in round 6.

Our estimates of the proportion of people with persistent COVID-19 symptoms are higher than in some other studies, although previous estimates have varied widely. At the low end, one study found that 2.3% of people with COVID-19 still reported symptoms at 12 weeks^8^; other studies have reported 13.7% of people were symptomatic at 84 days^9^, 14.8% symptomatic at 90 days^10^, 27% at 60 days^20^, 35% at 2 months^21^, 34.7% at 7 months^6^, 46% at 6 months^11^, and as high as 51–52% at 6 months^16,22^. Our estimates, that 37.7% of people with COVID-19 experience one or more symptoms at 12 weeks in autumn/winter 2020–2021, and 21.6% in spring 2021, may partly reflect the large list of symptoms we surveyed, many of which are common and not specific to COVID-19. However, the estimated background prevalence of persistent symptoms for 11+ days in more than 1.8 million PCR-negative REACT-1 respondents was ~3%, which provides an upper bound for non-COVID-19-related prevalence of persistent symptoms at 12 weeks or more. Our estimate of the prevalence of COVID-19-related persistent symptoms is therefore approximately tenfold the background prevalence. This is in agreement with a study of 26,922 UK residents between April and August 2021 by the Office for National Statistics (ONS), who estimated the point prevalence of any of 12 symptoms at 3.4% in non-COVID-19-positive people, with 0.5% reporting any symptom for 12 weeks or more^23^. Our estimate of the prevalence of COVID-19-related persistent symptoms is therefore approximately tenfold the background prevalence.

The overall reduction in persistent symptoms between rounds 3–5 and round 6 was driven by a decline in persistent tiredness of more than half, from 16.8% to 8.0%. There are several potential explanations. The majority (60%) of infections reported in round 6 were from pre-July 2020, so the decline in prevalence may reflect a proportion of people recovering from their illness and not reporting it (recall bias). Seasonality may affect symptom prevalence, although background symptom prevalence was largely consistent across the study period. Studies have found associations between lockdown measures and elevated levels of tiredness^24^ and stress^25,26^ and while rounds 3–5 were conducted predominantly when the UK was under restrictions or lockdown measures, round 6 was conducted during the transition from ‘step two’ of the reopening—when schools, retail and outdoor hospitality were open—to ‘step three’, when the ‘rule of six’ was implemented and indoor venues were allowed to reopen^27^. Finally, the structure of the survey changed in round 6, and the list of symptoms surveyed was amended. In our analysis of round 6, we focused on the 27 (of 29) symptoms that were in common with rounds 3–5, which may have slightly under-estimated symptom reporting prevalence in round 6 compared with the earlier rounds.

Increasing age, female sex, BMI, hospitalisation and co-morbidities have previously been identified as risk factors for Long COVID^8,28,29^. Our finding of a linear association between age and persistent symptoms following COVID-19 contrasts with some other studies that suggest the highest prevalence is found in middle-aged groups^9^. This discrepancy may reflect the fact that older age groups in the community have lower infection rates than younger people^30^ and are more likely to be asymptomatic^31,32^; once these factors were corrected for by conditioning on symptoms post-COVID-19, then the apparently lower prevalence of persistent symptoms at older ages was no longer seen.

Our identification of two stable symptom clusters at 12 weeks in rounds 3–5, with similar patterns identified in sensitivity analyses using different clustering methods, suggests that Long COVID may have distinct subgroups, including one (Cluster L2) characterised by high prevalence of shortness of breath and tight chest/chest pain. These and other related symptoms also had high prevalence in Cluster L2_R6_ in the round 6 replication data. Previous studies have taken a similar unsupervised approach to characterising subtypes of Long COVID, albeit at earlier time points: Sudre et al.^8^ identified two symptom clusters at 28 days post-symptom-onset, although these differed from our clusters. Huang et al.^20^ identified five clusters at 61 days, in two of which there was high prevalence of respiratory symptoms as seen in cluster L2 (and Cluster L2_R6_) in our study.

### Strengths and limitations

This study included data from a large random community sample with a high response rate (26–29% across 3–5), and use of weighting to provide population prevalence estimates, thus providing more representative information on persistent COVID-19 symptoms in the community. This is in contrast to other studies that have been based on specific patient groups, especially those based on hospitalised cases^5^. We asked about presence of symptoms rather than Long COVID to reduce potential reporting bias. However, it is clear that a wide spectrum of symptoms and clinical presentations post-COVID-19 may be involved; for example, our open free-text question identified a number of symptoms not included in our questionnaire including “brain fog”, “palpitations” and “hair loss”, which were subsequently included in round 6^33^. As the study was based on self-reported data and many of the symptoms are common and not specific to COVID-19, we compared our estimates with those obtained in the general population from people testing negative in the REACT-1 study.

Limitations include the retrospective study design, which introduces the possibility of recall bias. In previous analyses, however, we have shown that participant reports of date of onset of their symptoms produce an epidemic curve that very closely tracks the epidemic^31,34,35^. In addition, our analysis of REACT-1 data supports the finding of increasing proportions of asymptomatic infection in older age groups and suggests that this is not an artefact of differential recall of symptoms in older participants. Respondents were restricted to reporting a single date of (initial) symptom onset which does not allow for delayed onset of some symptoms, nor does it allow for the reporting of relapsing symptoms that appear to be a feature of Long COVID^8^. Respondents were also restricted to reporting overall illness severity, rather than symptom-specific severity, and were not asked to report when their symptoms were more severe. A further limitation, despite the high response rate, is the possibility of participation bias as the REACT-2 study included a self-administered LFIA^31^; it is plausible that people with persistent symptoms may have been more likely to participate in order to ascertain their antibody status.

### Implications

We have identified a substantial proportion of people who experience persistent symptoms lasting 12 weeks or more post COVID-19. After the initial decline in symptom prevalence between 4 and 12 weeks the prevalence of persistent symptoms plateaued indicating that large numbers of people may have chronic symptoms requiring investigation and intervention including rehabilitation. We show here that economically disadvantaged people and those in deprived areas appear to have a higher burden of persistent symptoms post COVID-19, compounding the excess burden of severe illness and mortality from COVID-19 experienced by these groups^36,37^.

We identified two clusters of participants based on their symptoms, including one in which shortness of breath and tight chest/chest pain predominated. Further studies are required to investigate the underlying pathophysiology. Clinicians and other healthcare professionals may benefit from education on the range of presenting symptoms to best support patients towards recovery.

In conclusion, the scale of morbidity identified in this study post COVID-19 presents significant challenges for the affected individuals and their families, and indicates a high potential population health burden. Managing the long-term sequelae of COVID-19 will remain a major challenge for affected individuals and their families and for health services.

## Methods

### Participants

The REACT-2 programme evaluated community prevalence of SARS-CoV-2 anti-spike protein antibody positivity in England. Random population samples of adults in England were invited to take part every 2–4 months using the National Health Service (NHS) patient list to achieve similar numbers of participants in each of 315 lower-tier local authority (LTLA) areas^38^. Participants registered via an online portal or by telephone. Those registered were sent a test kit by post that included a self-administered point-of-care LFIA test with instructions and a link to an online video. Participants completed a survey (online/telephone) upon completion of their self-test. Participants provided information on demographics, household composition, comorbidities, and whether or not they thought that they had had COVID-19. Those who reported having had COVID-19 were asked whether or not they had had a PCR test, symptoms related to COVID-19, date of first symptom onset, severity of symptoms, and duration of any of a list of 29 symptoms^39^. In addition, we asked participants to report any other symptoms in free text. Personalised invitations were sent to between 560,000 and 600,000 individuals aged 18+ years in each of rounds 3–5 of the REACT-2 study, carried out from 15 to 28 September 2020 (round 3), 27 October to 10 November 2020 (round 4) and 25 January to 8 February 2021 (round 5). Registrations closed after ~190,000 people had signed up at each round. A further 384,988 invitations were sent in round 6, carried out from 12 to 25 May 2021, and registration was closed after ~100,000 people had signed up. A booster sample of people aged 55 years and above was also recruited in round 6 but these data are excluded from analyses here for comparability with rounds 3–5.

Our primary study population comprised 76,155 participants from rounds 3–5 who self-reported having had COVID-19—either suspected or PCR confirmed—with one or more of 29 symptoms 12 weeks or more before the survey date (Supplementary Fig. 1). In addition to the 29 symptoms enquired about on the questionnaire in rounds 3–5, 8370 respondents gave free-text descriptions of other symptoms. Free-text analysis of co-occurring words indicated common additional symptoms which were not in the round 3–5 survey, including brain-fog, hair-loss, blood-pressure, heart-palpitations and, severe-joint-pain (Supplementary Fig. 3). Free text responses informed the additional symptoms that were surveyed in round 6 (35 symptoms in all of which 27—as 23 symptom groups—were in common with those asked in rounds 3–5).

We repeated our main analyses in an independent data set comprising 13,170 participants from round 6 who reported one or more of an expanded list of 35 symptoms 12 weeks or more before the survey date; 27 (as 23 symptom groups) of these 35 symptoms were in common with rounds 3–5 (see Supplementary Methods). To maintain comparability with symptom reporting in rounds 3–5 we also restricted some analyses in round 6 to the 27 symptoms in common.

In a sensitivity analysis we used a subset of 14,704 participants from rounds 3–5 who had a self-reported COVID-19 infection and tested positive for antibodies on the REACT-2 LFIA test.

To estimate background prevalence of symptoms, we used data from the REACT-1 study, which tracks community infection with PCR tests among independent population samples recruited with an identical sampling frame to REACT-2. REACT-1 also includes children aged 5–17 years, who were excluded from the current analyses. REACT-1 sought history of any of 26 symptoms that persisted for 11 or more days. The REACT-1 data were weighted in a similar fashion to the REACT-2 data to give population estimates that were representative of the adult population of England as a whole (see below).

### Data analysis

In rounds 3–5 (September 2020–February 2021) we obtained prevalence estimates for reporting of one or more of 29 symptoms by sex, age and other characteristics at 12 weeks after initial symptom onset. Our main analyses focused on individual symptoms reported as lasting for 12 weeks (84 days) or more, excluding 260 participants with inconsistent or missing data (see Fig. 1). We also obtained prevalence estimates for round 6 (May 2021).

Prevalence estimates were weighted by sex, age, ethnicity, LTLA population and index of multiple deprivation, to take account of the sampling design that gave approximately equal numbers of participants in each LTLA, and differential response rates, to obtain prevalence estimates that were representative of the population of England as a whole.

We used logistic regression (age-sex and mutually adjusted) to investigate the associations of demographic and lifestyle factors with persistence of symptoms at 12 weeks or more, and gradient boosted tree models^40^ to investigate predictive ability (area under the curve, AUC) changes from adding variables to the model for persistent symptoms at 12 weeks or more. This analysis was repeated in the REACT-2 round 6 data. Modelling approaches are described in detail in the Supplementary Methods.

To identify a more specific set of persistent symptoms associated with history of COVID-19, in sensitivity analyses, we carried out variable selection in a 30% subset of symptomatic participants in rounds 3–5: in univariable models, we identified a subset of persistent symptoms (12 or more weeks) that were positively associated with a reported prior positive PCR test and estimated the population prevalence of persistence of one or more of these symptoms. We also repeated the logistic and gradient boosted tree modelling with this subset of symptoms as outcome variables.

Generalised additive models (GAMs) were constructed with likelihood of symptom persistence at 12 weeks or more modelled as a smoothed function of age and sex. A default thin plate spline was used and the smoothed functions were plotted to visualise the relationship between risk of persistent symptoms and age.

We used the results from the free-text analysis to identify single and co-occurring words to indicate other symptoms recorded by participants and plotted these in a network.

To identify symptom clusters segmenting participants in rounds 3–5, a binary matrix was constructed for presence or absence (1 or 0) of each of the 29 surveyed symptoms at 12 weeks after symptom onset, for each participant. Clustering was performed using the CLustering LARge Applications (CLARA) extension of the Partitioning Around Medoids (PAM) algorithm, implemented in the R package fpc^41^. Briefly, PAM searches for the most representative data points to become cluster centroids by minimising the sum of dissimilarities between data points and their assigned centroids. CLARA uses a sampling approach to reduce the computational burden for large data sets. We used Hamming distance as a measure of dissimilarity between participants. In rounds 3–5, we determined the optimal number of clusters using the average silhouette width. We used two methods to assess cluster stability. First, we bootstrapped and re-clustered 100 times, then quantified the difference between bootstrapped and non-bootstrapped clusters using the Jaccard coefficient, which can range from 0 (no overlap) to 1 (perfect overlap)^42^. Second, we removed each symptom in turn, re-clustered, then calculated the average proportion of non-overlap (APN) between these and whole-dataset clusters as a proxy for the individual variable importance and contribution to the population segmentation^43^.

To visualise symptom patterns in the clusters we created heatmaps showing pairwise symptom co-occurrence at 12 weeks in the clusters separately.

As sensitivity analyses, we also ran PAM clustering using both Jaccard and Dice distance^44^ (which, unlike Hamming distance, do not consider negative cooccurrence), and, further, conducted Latent Class Analysis (LCA) as an entirely different approach to identifying structure in the symptom data. LCA was applied using the poLCA package in R^45^.

All data collection for the REACT2 study was captured with Questback (Spring 2020 installation)^46^. Analysis was conducted in R version 4.0.5^47^. We obtained research ethics approval from the South Central-Berkshire B Research Ethics Committee (IRAS ID: 283787). The REACT Public Advisory Panel provides regular review of the study processes and results. Participants in the study provided informed consent.

### Reporting summary

Further information on research design is available in the Nature Research Reporting Summary linked to this article.

## Supplementary information

Supplementary Information

Reporting Summary

## Data Availability

The original datasets generated or analysed, or both, during this study are not publicly available because of governance restrictions and the identifiable nature of the data. Requests for access to raw data should be addressed to the corresponding authors and will be answered within 12 weeks. Summary tabular data are provided here. The study materials and questionnaires used in this study can be found here.
