# Supplementary material for: Persistent COVID-19 symptoms in a community study of 606,434 people in England
Source: Nat Commun. 2022 Apr 12;13:1957. doi: 10.1038/s41467-022-29521-z (PMC9005552; doi:10.1038/s41467-022-29521-z)
Supplement: Supplementary file 1 — Supplementary Information [file 41467_2022_29521_MOESM1_ESM.pdf]

Persistent COVID-19 symptoms in a community study of 606,434 people in England: supplementary material

Whitaker M, Elliott J, Chadeau-Hyam et al, March 2022

Table of Contents

Supplementary figures and tables *3*

Table S1 Invitations and response rates. 4

Table S2 Prevalence of symptoms in rounds 3–5 and in round 6. 5

Table S3 Prevalence of persistent symptoms among people with symptomatic infections. 7

Table S4 Numbers and weighted prevalence of currently or previously reported persistent symptoms in the full REACT-2 study population (rounds 3–5). 13

Table S5 Round 6 replication of symptom persistence people with symptomatic infections. 19

Table S6 Round 6 replication of symptom persistence people with symptomatic infections (extended symptom list). 22

Table S7 Weighted prevalence of persistent symptoms in the population in round 6. 25

Table S8 Weighted prevalence of persistent symptoms in the population in round 6 (extended symptom set). 28

Table S9 Odds ratios for persistent symptoms at 12 weeks among symptomatic respondents. 31

Table S10 Characteristics of 12-week symptom clusters in rounds 3–5. 33

Table S11 Summary of persistent symptom prevalence among LFIA-positive individuals. 36

Table S12 Symptom prevalence in PCR-positive individuals in REACT-1. 37

Figure S1 Modelled probability of symptomatic infection and persistent symptoms, by age. 38

Figure S2 Modelling of persistent symptoms as a function of biological and demographic variables (round 6 replication). 39

Figure S3 Silhouette plot showing average silhouette width for 2 to 10 clusters. 40

Figure S4 Pairwise co-occurrence heatmaps of symptoms at 12 weeks after symptom onset, by cluster. 41

Figure S5 Visualisation of cluster separation and symptom contribution, using MCA. 42

Figure S6 Clustering results (round 6 replication). 43

Figure S7 Logistic modelling of PCR-positivity as a function of symptoms experienced. 44

Figure S8 Modelling of persistent symptoms as a function of biological and demographic variables (using 15-symptom subset). 45

Figure S9 Clustering results (with differing distance metrics). 46

Figure S13 Results of least absolute shrinkage and selection operator (LASSO) stability selection applied to predictors of persistent symptoms at 12 weeks among suspected or confirmed COVID-19. 50

Figure S14 Distribution of dates of self-reported COVID-19 illness.... 51

Supplementary methods....52

Modelling approaches 52

1. Logistic regression 52
2. Variable ranking and selection.... 52

Clustering: detailed methodology 53

Latent Class Analysis (LCA) 54

Replication analysis 55

Table S13 Details of symptoms surveyed in rounds 3–5 and in round 6 of REACT-2. 55

Survey (Rounds 3–5).... 56

Survey (Round 6) 57

Supplementary figures and tables

Table S1 Invitations and response rates.

Numbers invited, responders and non-responders, and percentages, by sex, age and Index of Multiple Deprivation (IMD) decile, and Chi-squared tests for heterogeneity, for REACT-2 (A) rounds 3–5 and (B) round 6.

A. Rounds 3–5

Variable	Category	All invitees	Responders	Non-responders	Percent among responders	Percent among non-responders	χ2 p-value
Sex	Female	868186	284840	583346	56.0	47.9	<0.001
	Male	857160	223861	633299	44.0	52.1	
Age	18 to 24	167506	28655	138851	5.6	11.4	<0.001
	25 to 34	304439	65159	239280	12.8	19.7	
	35 to 44	295640	85044	210596	16.7	17.3	
	45 to 54	294439	103478	190961	20.3	15.7	
	55 to 64	269967	105361	164606	20.7	13.5	
	65 to 74	211651	85297	126354	16.8	10.4	
	75 to 84	130143	31060	99083	6.1	8.1	
	85+	51561	4653	46908	0.9	3.9	
IMD decile	1 - most deprived	144116	21029	123087	4.1	10.1	<0.001
	2	167351	29231	138120	5.7	11.4	
	3	177489	36633	140856	7.2	11.6	
	4	179709	44261	135448	8.7	11.1	
	5	179754	51325	128429	10.1	10.6	
	6	183552	58603	124949	11.5	10.3	
	7	175658	60891	114767	12.0	9.4	
	8	177277	65346	111931	12.8	9.2	
	9	173076	68386	104690	13.4	8.6	
	10 - least deprived	167407	73002	94405	14.4	7.8	

B. Round 6

Variable	Category	All invitees	Responders	Non-responders	Percent among responders	Percent among non-responders	χ2 p-value
Sex	Female	193940	56871	137069	58.2	47.7	<0.001
	Male	191048	40853	150195	41.8	52.3	
Age	18 to 24	37102	4982	32120	5.1	11.2	<0.001
	25 to 34	67325	11791	55534	12.1	19.3	
	35 to 44	66662	15417	51245	15.8	17.8	
	45 to 54	65195	18980	46215	19.4	16.1	
	55 to 64	60845	20760	40085	21.2	14	
	65 to 74	47235	17770	29465	18.2	10.3	
	75 to 84	29188	7014	22174	7.2	7.7	
	85+	11436	1013	10423	1	3.6	
IMD decile	1 - most deprived	28255	4088	24167	4.2	12.7	<0.001
	2	32304	5597	26707	5.7	14.1	
	3	33332	7164	26168	7.3	13.8	
	4	31886	8683	23203	8.9	12.2	
	5	29960	9756	20204	10	10.7	
	6	29340	11233	18107	11.5	9.6	
	7	27147	11672	15475	11.9	8.2	
	8	26864	12498	14366	12.8	7.6	
	9	25395	13048	12347	13.4	6.5	
	10 - least deprived	22790	13988	8802	14.3	4.6	

Table S2 Prevalence of symptoms in rounds 3–5 and in round 6

The first 27 symptoms listed were surveyed in all rounds (asterisks indicate symptoms that were grouped in round 6). Chills and heavy arms/legs were surveyed in rounds 3–5 only, and the final 9 symptoms were surveyed in round 6 only.

Variable	Main analysis (Rounds 3–5, Sep 2020–Feb 2021)	Replication (Round 6, May 2021)	Sum over all rounds
Appetite loss	1942 (2.7%, [2.6-2.8])	93 (1%, [0.8-1.2])	2035
Nausea/vomiting*	600 (0.8%, [0.8-0.9])	158 (1.7%, [1.4-2])	758
Diarrhoea*	983 (1.4%, [1.3-1.4])	158 (1.7%, [1.4-2])	1141
Abdominal pain/belly ache*	1175 (1.6%, [1.5-1.7])	158 (1.7%, [1.4-2])	1333
Runny nose**	1882 (2.6%, [2.5-2.7])	154 (1.6%, [1.4-1.9])	2036
Sneezing	1512 (2.1%, [2-2.2])	64 (0.7%, [0.5-0.9])	1576
Blocked nose**	2102 (2.9%, [2.8-3])	154 (1.6%, [1.4-1.9])	2256
Sore eyes	2154 (3%, [2.8-3.1])	117 (1.2%, [1-1.5])	2271
Loss or change to sense of smell	3510 (4.8%, [4.7-5])	404 (4.3%, [3.9-4.7])	3914
Loss or change to sense of taste	2927 (4%, [3.9-4.2])	364 (3.9%, [3.5-4.3])	3291
Sore throat***	2212 (3%, [2.9-3.2])	172 (1.8%, [1.6-2.1])	2384
Hoarse voice***	1572 (2.2%, [2.1-2.3])	172 (1.8%, [1.6-2.1])	1744
Headache	3792 (5.2%, [5.1-5.4])	311 (3.3%, [3-3.7])	4103
Dizziness	2224 (3.1%, [2.9-3.2])	244 (2.6%, [2.3-2.9])	2468
Shortness of breath	7166 (9.8%, [9.6-10.1])	750 (7.9%, [7.4-8.5])	7916
New persistent cough	3073 (4.2%, [4.1-4.4])	361 (3.8%, [3.5-4.2])	3434
Tight chest****	4234 (5.8%, [5.7-6])	398 (4.2%, [3.8-4.6])	4632
Chest pain****	1854 (2.5%, [2.4-2.7])	398 (4.2%, [3.8-4.6])	2252
Fever	897 (1.2%, [1.2-1.3])	55 (0.6%, [0.4-0.8])	952
Difficulty sleeping	5427 (7.5%, [7.3-7.7])	438 (4.6%, [4.2-5.1])	5865
Tiredness	12214 (16.8%, [16.5-17.1])	759 (8%, [7.5-8.6])	12973
Severe fatigue	2098 (2.9%, [2.8-3])	234 (2.5%, [2.2-2.8])	2332
Numbness/tingling	1511 (2.1%, [2-2.2])	202 (2.1%, [1.9-2.5])	1713
Muscle aches	5264 (7.2%, [7-7.4])	391 (4.1%, [3.8-4.6])	5655
Red, itchy areas on the skin	794 (1.1%, [1-1.2])	179 (1.9%, [1.6-2.2])	973
Sudden swelling to face or lips	67 (0.1%, [0.1-0.1])	10 (0.1%, [0.1-0.2])	77
Purple sores/blisters on feet	221 (0.3%, [0.3-0.3])	34 (0.4%, [0.3-0.5])	255
Chills	906 (1.2%, [1.2-1.3])	-	906

Heavy arms/legs	2331 (3.2%, [3.1-3.3])	-	2331
Thrombosis	-	33 (0.3%, [0.2-0.5])	33
Confusion/brain fog/forgetfulness	-	590 (6.2%, [5.8-6.8])	590
Heart issues	-	287 (3%, [2.7-3.4])	287
Pain in joints	-	450 (4.8%, [4.4-5.2])	450
Weight loss	-	62 (0.7%, [0.5-0.8])	62
Vision issues	-	182 (1.9%, [1.7-2.2])	182
Hearing issues	-	196 (2.1%, [1.8-2.4])	196
Hair loss	-	131 (1.4%, [1.2-1.6])	131

Table S3 Prevalence of persistent symptoms among people with symptomatic infections.
Numbers and prevalence of persistent symptoms in the full REACT-2 study population (rounds 3–5), among the 76,155 symptomatic participants for whom we have 12 weeks' observation time and complete data. Table S2A shows prevalence of any of the 29 surveyed symptoms; Table S2B shows prevalence of any of a subset of 15 symptoms identified as predictive of PCR positivity. The final three columns show prevalence weighted to be representative of the adult population of England, by age, sex, ethnicity, lower-tier local authority population, and index of multiple deprivation (IMD).

(A) All 29 symptoms

			Counts at 12 weeks			*Prevalence at 12 weeks*		
**Variable**	**Category**	**n**	**Number with 1 or more symptoms at 12 weeks**	**Number with 2 or more symptoms at 12 weeks**	**Number with 3 or more symptoms at 12 weeks**	**Prevalence 1 or more symptoms at 12 weeks %**	**Prevalence 2 or more symptoms at 12 weeks %**	**Prevalence of 3 or more symptoms at 12 weeks %**
All participants	All participants	76155	28713	17860	11241	37.7 (37.4,38.0)	23.5 (23.2,23.8)	14.8 (14.5,15.0)
Sex	Male	32500	10604	6248	3728	32.6 (32.1,33.1)	19.2 (18.8,19.7)	11.5 (11.1,11.8)
	Female	43654	18109	11612	7513	41.5 (41.0,41.9)	26.6 (26.2,27.0)	17.2 (16.9,17.6)
Age group	18-24	5085	1534	885	514	30.2 (28.9,31.4)	17.4 (16.4,18.5)	10.1 (9.31,11.0)
	25-34	12245	3784	2105	1219	30.9 (30.1,31.7)	17.2 (16.5,17.9)	9.96 (9.44,10.5)
	35-44	15992	5232	3083	1815	32.7 (32.0,33.4)	19.3 (18.7,19.9)	11.3 (10.9,11.9)
	45-54	17548	6858	4311	2732	39.1 (38.4,39.8)	24.6 (23.9,25.2)	15.6 (15.0,16.1)
	55-64	15148	6461	4205	2713	42.7 (41.9,43.4)	27.8 (27.1,28.5)	17.9 (17.3,18.5)
	65-74	7811	3615	2406	1629	46.3 (45.2,47.4)	30.8 (29.8,31.8)	20.9 (20.0,21.8)
	74+	2326	1229	865	619	52.8 (50.8,54.9)	37.2 (35.2,39.2)	26.6 (24.9,28.4)
Ethnicity	Asian	2676	809	480	275	30.2 (28.5,32.0)	17.9 (16.5,19.4)	10.3 (9.18,11.5)
	Black	739	278	138	84	37.6 (34.2,41.2)	18.7 (16.0,21.6)	11.4 (9.28,13.9)
	Mixed	1244	487	310	202	39.1 (36.5,41.9)	24.9 (22.6,27.4)	16.2 (14.3,18.4)
	Other	793	299	180	118	37.7 (34.4,41.1)	22.7 (19.9,25.7)	14.9 (12.6,17.5)
	White	70103	26603	16600	10461	37.9 (37.6,38.3)	23.7 (23.4,24.0)	14.9 (14.7,15.2)
Adiposity	Underweight	886	333	220	147	37.6 (34.5,40.8)	24.8 (22.1,27.8)	16.6 (14.3,19.2)
	Normal weight	26128	8954	5325	3182	34.3 (33.7,34.8)	20.4 (19.9,20.9)	12.2 (11.8,12.6)
	Obese	23958	8916	5449	3398	37.2 (36.6,37.8)	22.7 (22.2,23.3)	14.2 (13.7,14.6)
	Overweight	16076	7107	4698	3127	44.2 (43.4,45.0)	29.2 (28.5,29.9)	19.5 (18.8,20.1)
Healthcare or care home worker	Yes	5243	2283	1469	920	43.5 (42.2,44.9)	28.0 (26.8,29.2)	17.5 (16.5,18.6)
Index of multiple	1 - most deprived	8096	3445	2288	1535	42.6 (41.5,43.6)	28.3 (27.3,29.3)	19.0 (18.1,19.8)

deprivation (IMD) quintile								
	2	12789	5066	3223	2118	39.6 (38.8,40.5)	25.2 (24.5,26.0)	16.6 (15.9,17.2)
	3	16491	6336	3937	2500	38.4 (37.7,39.2)	23.9 (23.2,24.5)	15.2 (14.6,15.7)
	4	18202	6728	4122	2553	37.0 (36.3,37.7)	22.6 (22.0,23.3)	14.0 (13.5,14.5)
	5 - least deprived	20577	7138	4290	2535	34.7 (34.0,35.3)	20.8 (20.3,21.4)	12.3 (11.9,12.8)
Current smoker	Current cigarette smoker	8441	3552	2367	1610	42.1 (41.0,43.1)	28.0 (27.1,29.0)	19.1 (18.2,19.9)
	Not current cigarette smoker	67021	24916	15344	9533	37.2 (36.8,37.5)	22.9 (22.6,23.2)	14.2 (14.0,14.5)
	Prefer not to say	693	245	149	98	35.4 (31.9,39.0)	21.5 (18.6,24.7)	14.1 (11.7,16.9)
Current vaper	Current vaper	4933	1986	1273	834	40.3 (38.9,41.6)	25.8 (24.6,27.0)	16.9 (15.9,18.0)
	Not current vaper	70802	26586	16493	10340	37.5 (37.2,37.9)	23.3 (23.0,23.6)	14.6 (14.3,14.9)
	Prefer not to say	420	141	94	67	33.6 (29.2,38.2)	22.4 (18.7,26.6)	16.0 (12.8,19.8)
Severity of COVID-19 symptoms	Mild symptoms	21454	5441	2746	1351	25.4 (24.8,25.9)	12.8 (12.4,13.3)	6.30 (5.98,6.63)
	Moderate symptoms	37976	14501	8841	5423	38.2 (37.7,38.7)	23.3 (22.9,23.7)	14.3 (13.9,14.6)
	Severe symptoms	16725	8771	6273	4467	52.4 (51.7,53.2)	37.5 (36.8,38.2)	26.7 (26.0,27.4)
Treatment sought/ received for COVID-19	No medical attention sought	47642	15098	8544	4912	31.7 (31.3,32.1)	17.9 (17.6,18.3)	10.3 (10.0,10.6)
	Sought medical attention from pharmacist / by phone (NHS 111/GP)	17794	8717	5991	4120	49.0 (48.3,49.7)	33.7 (33.0,34.4)	23.2 (22.5,23.8)
	Visited GP/walk-in centre/A&E	6154	2811	1911	1263	45.7 (44.4,46.9)	31.1 (29.9,32.2)	20.5 (19.5,21.6)
	Hospital admission	629	408	319	241	64.9 (61.1,68.5)	50.7 (46.8,54.6)	38.3 (34.6,42.2)
Gross household income	0-14,999	4543	2316	1673	1209	51.0 (49.5,52.4)	36.8 (35.4,38.2)	26.6 (25.3,27.9)
	15,000-49,999	21468	8746	5548	3521	40.7 (40.1,41.4)	25.8 (25.3,26.4)	16.4 (15.9,16.9)
	50,000-149,999	21923	7100	4088	2325	32.4 (31.8,33.0)	18.6 (18.1,19.2)	10.6 (10.2,11.0)
	>150,000	3082	886	463	245	28.7 (27.2,30.4)	15.0 (13.8,16.3)	7.95 (7.05,8.96)
Health conditions	Organ transplant recipient	70	35	27	20	50.0 (38.6,61.4)	38.6 (28.0,50.3)	28.6 (19.3,40.1)
	Diabetes (type I or II)	3282	1596	1118	837	48.6 (46.9,50.3)	34.1 (32.5,35.7)	25.5 (24.0,27.0)

Heart disease or heart problems	2822	1521	1090	816	53.9 (52.1,55.7)	38.6 (36.8,40.4)	28.9 (27.3,30.6)
Hypertension	8269	4009	2744	1905	48.5 (47.4,49.6)	33.2 (32.2,34.2)	23.0 (22.1,24.0)
Stroke	415	211	149	110	50.8 (46.0,55.6)	35.9 (31.4,40.6)	26.5 (22.5,31.0)
Kidney disease	547	325	238	166	59.4 (55.2,63.5)	43.5 (39.4,47.7)	30.3 (26.6,34.3)
Liver disease	309	171	123	103	55.3 (49.8,60.8)	39.8 (34.5,45.4)	33.3 (28.3,38.8)
Anaemia	1371	801	586	439	58.4 (55.8,61.0)	42.7 (40.1,45.4)	32.0 (29.6,34.5)
Asthma	9119	4419	3123	2192	48.5 (47.4,49.5)	34.2 (33.3,35.2)	24.0 (23.2,24.9)
Other lung condition	1639	1113	840	643	67.9 (65.6,70.1)	51.3 (48.8,53.7)	39.2 (36.9,41.6)
Cancer	820	411	270	196	50.1 (46.7,53.5)	32.9 (29.8,36.2)	23.9 (21.1,26.9)
Neurological condition	686	378	295	229	55.1 (51.4,58.8)	43.0 (39.3,46.7)	33.4 (30.0,37.0)
Immunocompromised*	3080	1815	1400	1055	58.9 (57.2,60.7)	45.5 (43.7,47.2)	34.3 (32.6,35.9)
Depression	6911	3931	2873	2146	56.9 (55.7,58.0)	41.6 (40.4,42.7)	31.1 (30.0,32.2)
Anxiety	10516	5553	3923	2809	52.8 (51.9,53.8)	37.3 (36.4,38.2)	26.7 (25.9,27.6)
Psychiatric disorder (other)	641	372	284	226	58.0 (54.2,61.8)	44.3 (40.5,48.2)	35.3 (31.7,39.0)

(B) 15 symptoms (loss or change of sense of smell, loss or change of sense of taste, tiredness, shortness of breath, heavy arms/legs, severe fatigue, dizziness, numbness/tingling, appetite loss, red, itchy areas on skin, difficulty sleeping, tight chest, headache, chest pain).

			Counts at 12 weeks			*Prevalence at 12 weeks*		
**Variable**	**Category**	**n**	**Number with 1 or more symptoms at 12 weeks**	**Number with 2 or more symptoms at 12 weeks**	**Number with 3 or more symptoms at 12 weeks**	**Prevalence 1 or more symptoms at 12 weeks %**	**Prevalence 2 or more symptoms at 12 weeks %**	**Prevalence of 3 or more symptoms at 12 weeks %**
All participants	All participants	76155	25052	14925	8663	32.9 (32.6,33.2)	19.6 (19.3,19.9)	11.4 (11.2,11.6)
Sex	Male	32500	9027	5134	2842	27.8 (27.3,28.3)	15.8 (15.4,16.2)	8.74 (8.44,9.06)
	Female	43654	16025	9791	5821	36.7 (36.3,37.2)	22.4 (22.0,22.8)	13.3 (13.0,13.7)
Age group	18-24	5085	1302	706	362	25.6 (24.4,26.8)	13.9 (13.0,14.9)	7.12 (6.44,7.86)
	25-34	12245	3230	1740	933	26.4 (25.6,27.2)	14.2 (13.6,14.8)	7.62 (7.16,8.10)
	35-44	15992	4586	2617	1409	28.7 (28.0,29.4)	16.4 (15.8,16.9)	8.81 (8.38,9.26)
	45-54	17548	6087	3733	2225	34.7 (34.0,35.4)	21.3 (20.7,21.9)	12.7 (12.2,13.2)
	55-64	15148	5658	3488	2099	37.4 (36.6,38.1)	23.0 (22.4,23.7)	13.9 (13.3,14.4)
	65-74	7811	3121	1942	1194	40.0 (38.9,41.0)	24.9 (23.9,25.8)	15.3 (14.5,16.1)
	74+	2326	1068	699	441	45.9 (43.9,47.9)	30.1 (28.2,31.9)	19.0 (17.4,20.6)
Ethnicity	Asian	2676	692	395	217	25.9 (24.2,27.6)	14.8 (13.5,16.2)	8.11 (7.13,9.20)
	Black	739	236	118	65	31.9 (28.7,35.4)	16.0 (13.5,18.8)	8.80 (6.96,11.1)
	Mixed	1244	427	265	170	34.3 (31.7,37.0)	21.3 (19.1,23.7)	13.7 (11.9,15.7)
	Other	793	254	142	92	32.0 (28.9,35.4)	17.9 (15.4,20.7)	11.6 (9.56,14.0)
	White	70103	23233	13874	8038	33.1 (32.8,33.5)	19.8 (19.5,20.1)	11.5 (11.2,11.7)
Adiposity	Underweight	886	295	183	122	33.3 (30.3,36.5)	20.7 (18.1,23.4)	13.8 (11.7,16.2)
	Normal weight	26128	7706	4334	2371	29.5 (28.9,30.0)	16.6 (16.1,17.0)	9.07 (8.73,9.43)
	Obese	23958	7726	4539	2629	32.2 (31.7,32.8)	18.9 (18.5,19.4)	11.0 (10.6,11.4)
	Overweight	16076	6346	4038	2467	39.5 (38.7,40.2)	25.1 (24.5,25.8)	15.3 (14.8,15.9)
Healthcare or care home worker	Yes	5243	2088	1298	764	39.8 (38.5,41.2)	24.8 (23.6,25.9)	14.6 (13.6,15.6)
Index of multiple deprivation (IMD) quintile	1 - most deprived	8096	3073	1955	1211	38.0 (36.9,39.0)	24.1 (23.2,25.1)	15.0 (14.2,15.8)
	2	12789	4453	2699	1675	34.8 (34.0,35.6)	21.1 (20.4,21.8)	13.1 (12.5,13.7)
	3	16491	5505	3324	1973	33.4 (32.7,34.1)	20.2 (19.6,20.8)	12.0 (11.5,12.5)

	4	18202	5858	3427	1926	32.2 (31.5,32.9)	18.8 (18.3,19.4)	10.6 (10.1,11.0)
	5 - least deprived	20577	6163	3520	1878	30.0 (29.3,30.6)	17.1 (16.6,17.6)	9.13 (8.74,9.53)
Current smoker	Current cigarette smoker	8441	3120	2011	1256	37.0 (35.9,38.0)	23.8 (22.9,24.7)	14.9 (14.1,15.7)
	Not current cigarette smoker	67021	21715	12785	7329	32.4 (32.0,32.8)	19.1 (18.8,19.4)	10.9 (10.7,11.2)
	Prefer not to say	693	217	129	78	31.3 (28.0,34.9)	18.6 (15.9,21.7)	11.3 (9.11,13.8)
Current vaper	Current vaper	4933	1762	1085	655	35.7 (34.4,37.1)	22.0 (20.9,23.2)	13.3 (12.4,14.3)
	Not current vaper	70802	23160	13757	7955	32.7 (32.4,33.1)	19.4 (19.1,19.7)	11.2 (11.0,11.5)
	Prefer not to say	420	130	83	53	31.0 (26.7,35.5)	19.8 (16.2,23.8)	12.6 (9.78,16.1)
Severity of COVID-19 symptoms	Mild symptoms	21454	4378	2087	838	20.4 (19.9,21.0)	9.73 (9.34,10.1)	3.91 (3.65,4.17)
	Moderate symptoms	37976	12626	7287	4114	33.2 (32.8,33.7)	19.2 (18.8,19.6)	10.8 (10.5,11.1)
	Severe symptoms	16725	8048	5551	3711	48.1 (47.4,48.9)	33.2 (32.5,33.9)	22.2 (21.6,22.8)
Treatment sought/ received for COVID-19	No medical attention sought	47642	12864	6963	3571	27.0 (26.6,27.4)	14.6 (14.3,14.9)	7.50 (7.26,7.74)
	Sought medical attention from pharmacist / by phone (NHS 111/GP)	17794	7833	5161	3369	44.0 (43.3,44.8)	29.0 (28.3,29.7)	18.9 (18.4,19.5)
	Visited GP/walk-in centre/A&E	6154	2477	1595	966	40.3 (39.0,41.5)	25.9 (24.8,27.0)	15.7 (14.8,16.6)
	Hospital admission	629	393	293	209	62.5 (58.6,66.2)	46.6 (42.7,50.5)	33.2 (29.7,37.0)
Gross household income	0-14,999	4543	2079	1396	944	45.8 (44.3,47.2)	30.7 (29.4,32.1)	20.8 (19.6,22.0)
	15,000-49,999	21468	7656	4665	2729	35.7 (35.0,36.3)	21.7 (21.2,22.3)	12.7 (12.3,13.2)
	50,000-149,999	21923	6129	3389	1775	28.0 (27.4,28.6)	15.5 (15.0,15.9)	8.10 (7.74,8.46)
	>150,000	3082	757	387	183	24.6 (23.1,26.1)	12.6 (11.4,13.8)	5.94 (5.16,6.83)
Health conditions	Organ transplant recipient	70	30	23	16	42.9 (31.9,54.5)	32.9 (23.0,44.5)	22.9 (14.6,34.0)
	Diabetes (type I or II)	3282	1425	965	664	43.4 (41.7,45.1)	29.4 (27.9,31.0)	20.2 (18.9,21.6)
	Heart disease or heart problems	2822	1357	934	655	48.1 (46.2,49.9)	33.1 (31.4,34.9)	23.2 (21.7,24.8)
	Hypertension	8269	3555	2326	1485	43.0 (41.9,44.1)	28.1 (27.2,29.1)	18.0 (17.1,18.8)
	Stroke	415	191	132	97	46.0 (41.3,50.8)	31.8 (27.5,36.4)	23.4 (19.6,27.7)

Kidney disease	547	295	208	133	53.9 (49.7,58.1)	38.0 (34.1,42.2)	24.3 (20.9,28.1)
Liver disease	309	153	110	81	49.5 (44.0,55.1)	35.6 (30.5,41.1)	26.2 (21.6,31.4)
Anaemia	1371	737	522	370	53.8 (51.1,56.4)	38.1 (35.5,40.7)	27.0 (24.7,29.4)
Asthma	9119	4042	2741	1775	44.3 (43.3,45.3)	30.1 (29.1,31.0)	19.5 (18.7,20.3)
Other lung condition	1639	1025	740	553	62.5 (60.2,64.8)	45.1 (42.8,47.6)	33.7 (31.5,36.1)
Cancer	820	351	229	157	42.8 (39.5,46.2)	27.9 (25.0,31.1)	19.1 (16.6,22.0)
Neurological condition	686	351	271	203	51.2 (47.4,54.9)	39.5 (35.9,43.2)	29.6 (26.3,33.1)
Immunocompromised*	3080	1662	1235	882	54.0 (52.2,55.7)	40.1 (38.4,41.8)	28.6 (27.1,30.3)
Depression	6911	3645	2546	1771	52.7 (51.6,53.9)	36.8 (35.7,38.0)	25.6 (24.6,26.7)
Anxiety	10516	5096	3449	2275	48.5 (47.5,49.4)	32.8 (31.9,33.7)	21.6 (20.9,22.4)
Psychiatric disorder (other)	641	347	251	190	54.1 (50.3,58.0)	39.2 (35.5,43.0)	29.6 (26.2,33.3)

Table S4 Numbers and weighted prevalence of currently or previously reported persistent symptoms in the full REACT-2 study population (rounds 3–5).
Table 1A shows prevalence of any of the 29 surveyed symptoms; Table 1B shows prevalence of any of a subset of 15 symptoms identified as predictive of PCR positivity. The final three columns show prevalence weighted to be representative of the adult population of England, by age, sex, ethnicity, lower-tier local authority population, and index of multiple deprivation (IMD).

(A) All 29 symptoms

			Counts at 12 weeks			*Prevalence at 12 weeks*		
**Variable**	**Category**	**n**	**Number with 1 or more symptoms**	**Number with 2 or more symptoms**	**Number with 3 or more symptoms**	**Prevalence 1 or more symptoms (weighted) %**	**Prevalence 2 or more symptoms (weighted) %**	**Prevalence 3 or more symptoms (weighted) %**
All participants		508707	28973	17966	11287	5.80 (5.73,5.86)	3.56 (3.51,3.61)	2.23 (2.19,2.27)
Sex	Women	223861	10697	6284	3746	4.94 (4.86,5.03)	2.86 (2.80,2.93)	1.69 (1.64,1.74)
	Men	284840	18276	11682	7541	6.62 (6.52,6.71)	4.23 (4.15,4.30)	2.75 (2.68,2.81)
Age group	18-24	28655	1535	886	514	5.65 (5.46,5.85)	3.19 (3.05,3.34)	1.84 (1.73,1.95)
	25-34	65159	3795	2110	1221	6.05 (5.89,6.21)	3.34 (3.22,3.46)	1.88 (1.79,1.97)
	35-44	85044	5276	3106	1824	6.25 (6.08,6.42)	3.65 (3.53,3.79)	2.15 (2.05,2.25)
	45-54	103478	6927	4338	2740	6.86 (6.69,7.02)	4.31 (4.18,4.45)	2.73 (2.63,2.84)
	55-64	105361	6536	4235	2731	6.39 (6.22,6.56)	4.19 (4.05,4.34)	2.75 (2.64,2.87)
	65-74	85297	3661	2420	1635	4.61 (4.45,4.77)	3.07 (2.94,3.20)	2.09 (1.99,2.21)
	74+	35713	1243	871	622	3.68 (3.52,3.84)	2.60 (2.47,2.73)	1.92 (1.81,2.04)
Ethnicity	Asian	17666	823	487	279	5.13 (4.91,5.36)	2.83 (2.66,3.00)	1.60 (1.48,1.73)
	Black	4091	285	141	86	6.75 (6.33,7.18)	3.53 (3.23,3.86)	2.07 (1.84,2.32)
	Mixed	6112	490	312	202	7.70 (7.04,8.42)	4.73 (4.21,5.31)	3.05 (2.63,3.53)
	Other	4434	306	185	121	6.82 (6.18,7.51)	4.17 (3.67,4.73)	2.81 (2.41,3.28)
	White	472750	26826	16688	10497	5.78 (5.71,5.85)	3.59 (3.54,3.65)	2.27 (2.22,2.31)
Adiposity	Underweight	6273	336	220	147	5.93 (5.42,6.48)	3.70 (3.30,4.14)	2.14 (1.84,2.49)
	Normal weight	183305	9041	5359	3201	5.08 (4.98,5.18)	2.97 (2.90,3.05)	1.76 (1.70,1.82)
	Obese	161456	8987	5479	3407	5.76 (5.65,5.88)	3.50 (3.41,3.59)	2.19 (2.12,2.26)
	Overweight	97597	7177	4725	3136	7.36 (7.19,7.52)	4.72 (4.59,4.85)	3.16 (3.05,3.27)
Healthcare or care home worker	Yes	27690	2311	1483	925	8.55 (8.22,8.88)	5.31 (5.05,5.58)	3.22 (3.01,3.43)
Index of multiple	1 - most deprived	50260	3477	2299	1538	6.85 (6.70,7.01)	4.41 (4.28,4.54)	2.90 (2.80,3.01)

deprivation (IMD) quintile	2	80894	5116	3248	2129	6.28 (6.14,6.43)	3.84 (3.72,3.95)	2.48 (2.39,2.57)
	3	109928	6390	3961	2512	5.59 (5.46,5.74)	3.41 (3.30,3.52)	2.11 (2.03,2.20)
	4	126237	6792	4149	2563	5.28 (5.14,5.42)	3.18 (3.07,3.29)	1.93 (1.85,2.02)
	5 - least deprived	141388	7198	4309	2545	4.96 (4.83,5.10)	2.96 (2.86,3.07)	1.73 (1.65,1.81)
Current smoker	Current cigarette smoker	50983	3569	2372	1613	7.05 (6.84,7.26)	4.60 (4.44,4.78)	3.11 (2.97,3.26)
	Not current cigarette smoker	453681	25154	15442	9573	5.63 (5.57,5.70)	3.42 (3.37,3.47)	2.12 (2.07,2.16)
	Prefer not to say	4043	250	152	101	6.17 (5.50,6.91)	4.04 (3.50,4.65)	2.42 (2.01,2.91)
Current vaper	Current vaper	28436	1997	1276	834	7.24 (6.95,7.53)	4.59 (4.36,4.83)	2.93 (2.75,3.13)
	Not current vaper	477729	26834	16596	10386	5.70 (5.64,5.77)	3.49 (3.44,3.54)	2.18 (2.14,2.22)
	Prefer not to say	2542	142	94	67	5.61 (4.83,6.51)	3.95 (3.30,4.72)	2.43 (1.92,3.05)
Severity of COVID symptoms	Mild symptoms	27572	5478	2757	1354	18.7 (18.3,19.1)	9.29 (8.97,9.62)	4.48 (4.25,4.71)
	Moderate symptoms	45357	14618	8885	5440	31.4 (31.0,31.8)	18.8 (18.5,19.2)	11.6 (11.3,11.9)
	Severe symptoms	19187	8877	6324	4493	45.0 (44.3,45.7)	32.1 (31.4,32.7)	22.7 (22.2,23.3)
Treatment sought/ received for COVID	No medical attention sought	58330	15203	8585	4929	24.9 (24.5,25.2)	13.9 (13.6,14.2)	7.95 (7.74,8.17)
	Sought medical attention from pharmacist / by phone (NHS 111/GP)	21388	8809	6033	4140	39.6 (38.9,40.2)	26.6 (26.1,27.2)	18.4 (17.9,18.9)
	Visited GP/walk-in centre/A&E	6792	2855	1930	1271	40.4 (39.2,41.6)	27.9 (26.8,29.0)	18.1 (17.2,19.0)
		739	413	320	242	53.9 (50.5,57.2)	41.5 (38.2,44.8)	31.1 (28.1,34.3)
	Hospital admission	32819	2346	1685	1213	7.20 (6.94,7.46)	5.09 (4.87,5.32)	3.72 (3.53,3.91)
Gross household income	0-14,999	148039	8819	5580	3536	6.11 (5.99,6.23)	3.84 (3.75,3.94)	2.39 (2.32,2.47)
	15,000-49,999	128957	7156	4109	2334	5.69 (5.56,5.82)	3.17 (3.07,3.27)	1.78 (1.71,1.85)
	50,000-149,999	16279	895	466	248	5.60 (5.23,5.99)	2.73 (2.48,3.01)	1.45 (1.27,1.66)
		596	36	27	20	5.71 (4.12,7.88)	4.20 (2.86,6.13)	3.53 (2.32,5.34)
	>150,000	27358	1617	1126	841	5.98 (5.71,6.26)	4.06 (3.83,4.29)	3.14 (2.94,3.35)
Comorbidities	Organ transplant recipient	21430	1535	1098	819	7.15 (6.82,7.50)	5.13 (4.84,5.43)	3.86 (3.61,4.13)

Diabetes (type I or II)	65792	4048	2762	1915	6.22 (6.04,6.41)	4.27 (4.12,4.43)	3.03 (2.89,3.16)
Heart disease or heart problems	3352	214	149	110	6.23 (5.48,7.08)	4.15 (3.53,4.86)	3.14 (2.62,3.78)
Hypertension (high blood pressure)	3867	326	239	166	8.40 (7.58,9.30)	6.11 (5.40,6.90)	4.11 (3.54,4.78)
Stroke	2107	175	126	104	8.51 (7.41,9.75)	5.81 (4.90,6.87)	4.76 (3.94,5.73)
Kidney disease	7575	807	588	439	11.1 (10.5,11.8)	7.83 (7.28,8.42)	5.78 (5.30,6.29)
Liver disease	51337	4461	3137	2196	8.78 (8.54,9.03)	6.09 (5.88,6.29)	4.22 (4.06,4.40)
Anaemia	12500	1122	842	643	9.07 (8.59,9.57)	6.74 (6.32,7.18)	5.32 (4.95,5.72)
Asthma	8042	412	270	196	5.22 (4.74,5.73)	3.49 (3.10,3.92)	2.65 (2.32,3.03)
Other lung condition	5415	380	296	229	7.12 (6.47,7.84)	5.38 (4.81,6.02)	4.07 (3.58,4.63)
Cancer	19302	1832	1407	1059	9.75 (9.33,10.2)	7.51 (7.14,7.89)	5.63 (5.30,5.97)
Condition affecting the brain and nerves	38125	3966	2885	2151	10.4 (10.1,10.7)	7.58 (7.33,7.84)	5.59 (5.37,5.82)
A weakened immune system/reduced ability to deal with infections	57140	5602	3948	2818	10.1 (9.87,10.3)	7.08 (6.88,7.29)	5.04 (4.86,5.21)
Depression	3385	374	285	226	10.8 (9.90,11.8)	8.11 (7.30,9.00)	6.13 (5.42,6.92)
Anxiety	508707	28973	17966	11287	4.95 (4.88,5.01)	3.03 (2.98,3.08)	1.93 (1.89,1.97)
Psychiatric disorder	223861	10697	6284	3746	17.8 (17.4,18.2)	11.0 (10.7,11.4)	6.57 (6.32,6.82)

(B) 15 symptoms (loss or change of sense of smell, loss or change of sense of taste, tiredness, shortness of breath, heavy arms/legs, severe fatigue, dizziness, numbness/tingling, appetite loss, red, itchy areas on skin, difficulty sleeping, tight chest, headache, chest pain).

			Counts at 12 weeks			*Prevalence at 12 weeks*		
**Variable**	**Category**	**n**	**Number with 1 or more symptoms**	**Number with 2 or more symptoms**	**Number with 3 or more symptoms**	**Prevalence 1 or more symptoms (weighted) %**	**Prevalence 2 or more symptoms (weighted) %**	**Prevalence 3 or more symptoms (weighted) %**
All participants		508707	25266	14999	8694	5.03 (4.97,5.09)	2.98 (2.93,3.02)	1.71 (1.68,1.75)
Sex	Women	223861	9103	5162	2856	4.19 (4.11,4.27)	2.36 (2.30,2.42)	1.30 (1.25,1.34)
	Men	284840	16163	9837	5838	5.83 (5.74,5.93)	3.57 (3.50,3.64)	2.11 (2.06,2.17)
Age group	18-24	28655	1303	707	362	4.67 (4.49,4.85)	2.58 (2.45,2.71)	1.35 (1.25,1.45)
	25-34	65159	3240	1743	933	5.17 (5.02,5.31)	2.75 (2.65,2.86)	1.41 (1.33,1.49)
	35-44	85044	4622	2632	1416	5.42 (5.27,5.58)	3.12 (3.00,3.24)	1.69 (1.60,1.78)
	45-54	103478	6144	3754	2230	6.09 (5.94,6.25)	3.72 (3.60,3.85)	2.22 (2.13,2.32)
	55-64	105361	5715	3511	2109	5.60 (5.44,5.76)	3.48 (3.35,3.61)	2.13 (2.03,2.24)
	65-74	85297	3161	1950	1200	3.99 (3.84,4.14)	2.49 (2.37,2.61)	1.55 (1.46,1.65)
	74+	35713	1081	702	444	3.25 (3.11,3.41)	2.16 (2.04,2.28)	1.39 (1.29,1.49)
Ethnicity	Asian	17666	704	401	220	4.24 (4.04,4.46)	2.38 (2.23,2.54)	1.26 (1.15,1.38)
	Black	4091	243	120	66	5.61 (5.23,6.02)	3.00 (2.72,3.30)	1.63 (1.43,1.86)
	Mixed	6112	429	266	170	6.67 (6.05,7.35)	3.90 (3.43,4.44)	2.36 (2.00,2.79)
	Other	4434	260	147	94	5.78 (5.20,6.43)	3.28 (2.84,3.78)	2.16 (1.81,2.58)
	White	472750	23415	13933	8062	5.04 (4.97,5.10)	3.00 (2.95,3.05)	1.74 (1.70,1.78)
ADIPOSITY	Underweight	6273	298	183	122	5.07 (4.60,5.59)	3.00 (2.64,3.40)	1.69 (1.42,2.00)
	Normal weight	183305	7784	4359	2385	4.38 (4.29,4.47)	2.44 (2.37,2.51)	1.32 (1.27,1.38)
	Overweight	161456	7783	4563	2637	4.95 (4.85,5.06)	2.93 (2.84,3.01)	1.69 (1.63,1.76)
	Obese	97597	6399	4054	2473	6.51 (6.35,6.67)	4.05 (3.93,4.18)	2.48 (2.38,2.58)
Healthcare or care home worker	Yes	27690	2114	1308	768	7.58 (7.27,7.90)	4.70 (4.46,4.96)	2.66 (2.47,2.86)
Index of multiple deprivation (IMD) quintile	1 - most deprived	50260	3098	1962	1213	6.00 (5.85,6.15)	3.75 (3.64,3.87)	2.28 (2.19,2.37)
	2	80894	4492	2715	1684	5.51 (5.37,5.65)	3.21 (3.11,3.32)	1.92 (1.84,2.01)
	3	109928	5553	3340	1978	4.80 (4.68,4.93)	2.87 (2.77,2.97)	1.65 (1.58,1.73)
	4	126237	5910	3444	1935	4.55 (4.43,4.68)	2.62 (2.53,2.73)	1.44 (1.37,1.52)

	5 - least deprived	141388	6213	3538	1884	4.28 (4.15,4.41)	2.42 (2.33,2.52)	1.28 (1.21,1.35)
Current smoker	Current cigarette smoker	50983	3132	2015	1258	6.16 (5.97,6.36)	3.87 (3.72,4.04)	2.41 (2.29,2.54)
	Not current cigarette smoker	453681	21912	12853	7356	4.88 (4.82,4.94)	2.86 (2.81,2.90)	1.62 (1.59,1.66)
	Prefer not to say	4043	222	131	80	5.57 (4.94,6.28)	3.53 (3.03,4.11)	1.91 (1.55,2.35)
Current vaper	Current vaper	28436	1769	1086	655	6.39 (6.13,6.67)	3.98 (3.77,4.20)	2.27 (2.11,2.44)
	Not current vaper	477729	23366	13830	7986	4.94 (4.88,5.00)	2.91 (2.86,2.96)	1.68 (1.64,1.72)
	Prefer not to say	2542	131	83	53	5.23 (4.48,6.11)	3.33 (2.73,4.05)	1.77 (1.35,2.32)
Severity of COVID symptoms	Mild symptoms	27572	4407	2094	840	14.9 (14.6,15.4)	7.03 (6.75,7.32)	2.79 (2.61,2.98)
	Moderate symptoms	45357	12718	7314	4126	27.1 (26.7,27.5)	15.5 (15.2,15.9)	8.85 (8.59,9.10)
	Severe symptoms	19187	8141	5591	3728	41.2 (40.5,41.9)	28.5 (27.9,29.1)	18.6 (18.1,19.2)
Treatment sought/ received for COVID	No medical attention sought	58330	12950	6992	3584	21.0 (20.7,21.4)	11.3 (11.0,11.5)	5.82 (5.63,6.00)
	Sought medical attention from pharmacist / by phone (NHS 111/GP)	21388	7910	5192	3381	35.4 (34.8,36.0)	23.0 (22.5,23.6)	14.9 (14.5,15.4)
	Visited GP/walk-in centre/A&E	6792	2512	1607	971	35.6 (34.5,36.8)	23.4 (22.4,24.4)	13.5 (12.7,14.3)
	Hospital admission	739	397	294	210	51.6 (48.2,55.0)	38.5 (35.3,41.8)	27.2 (24.3,30.3)
Gross household income	0-14,999	32819	2102	1406	948	6.38 (6.13,6.63)	4.22 (4.02,4.43)	2.90 (2.74,3.08)
	15,000-49,999	148039	7720	4687	2738	5.34 (5.23,5.46)	3.24 (3.15,3.33)	1.87 (1.80,1.94)
	50,000-149,999	128957	6179	3405	1781	4.88 (4.76,5.00)	2.64 (2.55,2.73)	1.35 (1.29,1.41)
	>150,000	16279	766	389	184	4.73 (4.39,5.09)	2.29 (2.06,2.55)	1.03 (0.878,1.21)
Comorbidities	Organ transplant recipient	596	31	23	16	4.55 (3.14,6.53)	3.70 (2.45,5.53)	2.86 (1.79,4.53)
	Diabetes (type I or II)	27358	1443	970	668	5.29 (5.04,5.56)	3.52 (3.31,3.74)	2.54 (2.36,2.73)
	Heart disease or heart problems	21430	1370	936	656	6.45 (6.13,6.79)	4.35 (4.09,4.63)	3.03 (2.81,3.27)
	Hypertension (high	65792	3589	2337	1489	5.55 (5.37,5.73)	3.66 (3.52,3.81)	2.40 (2.28,2.52)

blood pressure)							
Stroke	3352	194	132	97	5.66 (4.94,6.48)	3.60 (3.03,4.27)	2.75 (2.25,3.34)
Kidney disease	3867	296	208	133	7.80 (7.00,8.67)	5.00 (4.36,5.72)	3.31 (2.79,3.91)
Liver disease	2107	157	111	82	7.73 (6.68,8.92)	4.98 (4.15,5.98)	3.61 (2.91,4.48)
Anaemia	7575	741	522	370	10.1 (9.46,10.7)	6.80 (6.29,7.35)	4.76 (4.33,5.23)
Asthma	51337	4075	2748	1777	7.94 (7.71,8.17)	5.31 (5.12,5.51)	3.38 (3.23,3.54)
Other lung condition	12500	1032	741	553	8.34 (7.88,8.83)	5.97 (5.58,6.39)	4.56 (4.22,4.94)
Cancer	8042	352	229	157	4.50 (4.06,4.98)	2.93 (2.58,3.33)	2.19 (1.89,2.54)
Condition affecting the brain and nerves	5415	352	271	203	6.59 (5.96,7.28)	4.94 (4.39,5.55)	3.59 (3.13,4.12)
A weakened immune system/reduced ability to deal with infections	19302	1675	1240	884	8.95 (8.55,9.37)	6.70 (6.34,7.06)	4.68 (4.38,4.99)
Depression	38125	3673	2554	1773	9.63 (9.35,9.91)	6.77 (6.53,7.02)	4.62 (4.42,4.82)
Anxiety	57140	5137	3464	2281	9.22 (9.00,9.46)	6.25 (6.06,6.44)	4.06 (3.91,4.22)
Psychiatric disorder	3385	349	252	190	9.91 (9.02,10.9)	6.94 (6.19,7.78)	5.24 (4.58,5.98)

Table S5 Round 6 replication of symptom persistence people with symptomatic infections.

Numbers and proportions of participants who reported one or more symptoms (from a list of 27* symptoms surveyed in all four rounds) of COVID-19 at 12 weeks post symptom onset, among the 13,170 symptomatic participants from REACT-2 round 6 for whom we have 12 weeks' observation time and complete data.

			Counts at 12 weeks			*Prevalence at 12 weeks*		
**Variable**	**Category**	**n**	**Number with 1 or more symptoms**	**Number with 2 or more symptoms**	**Number with 3 or more symptoms**	**Prevalence 1 or more symptoms (unweighted)**	**Prevalence 2 or more symptoms (unweighted)**	**Prevalence 3 or more symptoms (unweighted)**
All participants	All participants	13170	2845	2188	1572	21.6 (20.9,22.3)	16.6 (16.0,17.3)	11.9 (11.4,12.5)
Sex	Male	5100	892	665	452	17.5 (16.5,18.6)	13.0 (12.1,14.0)	8.86 (8.11,9.67)
	Female	8069	1953	1523	1120	24.2 (23.3,25.2)	18.9 (18.0,19.7)	13.9 (13.1,14.7)
Age group	18-24	968	110	80	53	11.4 (9.52,13.5)	8.26 (6.69,10.2)	5.48 (4.21,7.09)
	25-34	2266	348	248	153	15.4 (13.9,16.9)	10.9 (9.72,12.3)	6.75 (5.79,7.86)
	35-44	2818	508	391	268	18.0 (16.7,19.5)	13.9 (12.6,15.2)	9.51 (8.48,10.6)
	45-54	3022	719	548	401	23.8 (22.3,25.3)	18.1 (16.8,19.5)	13.3 (12.1,14.5)
	55-64	2520	724	571	420	28.7 (27.0,30.5)	22.7 (21.1,24.3)	16.7 (15.3,18.2)
	65-74	1197	321	257	201	26.8 (24.4,29.4)	21.5 (19.2,23.9)	16.8 (14.8,19.0)
	74+	379	115	93	76	30.3 (25.9,35.1)	24.5 (20.5,29.1)	20.1 (16.3,24.4)
Ethnicity	Asian	590	88	61	47	14.9 (12.3,18.0)	10.3 (8.13,13.1)	7.97 (6.04,10.4)
	Black	204	43	33	24	21.1 (16.0,27.2)	16.2 (11.8,21.8)	11.8 (8.03,16.9)
	Mixed	245	50	35	29	20.4 (15.8,25.9)	14.3 (10.5,19.2)	11.8 (8.37,16.5)
	Other	173	39	26	18	22.5 (17.0,29.3)	15.0 (10.5,21.1)	10.4 (6.68,15.8)
	White	11836	2593	2006	1432	21.9 (21.2,22.7)	16.9 (16.3,17.6)	12.1 (11.5,12.7)
Adiposity	Underweight	163	36	23	14	22.1 (16.4,29.1)	14.1 (9.59,20.3)	8.59 (5.19,13.9)
	Normal weight	4511	822	597	411	18.2 (17.1,19.4)	13.2 (12.3,14.3)	9.11 (8.31,9.99)
	Overweight	4028	816	634	433	20.3 (19.0,21.5)	15.7 (14.6,16.9)	10.7 (9.83,11.7)
	Obese	2816	802	652	493	28.5 (26.8,30.2)	23.2 (21.6,24.7)	17.5 (16.1,19.0)
Healthcare or care home worker	Yes	1342	366	278	193	27.3 (25.0,29.7)	20.7 (18.6,23.0)	14.4 (12.6,16.4)
Index of multiple deprivation (IMD) quintile	1 - most deprived	1480	344	280	214	23.2 (21.2,25.5)	18.9 (17.0,21.0)	14.5 (12.8,16.3)
	2	2374	521	405	281	21.9 (20.3,23.7)	17.1 (15.6,18.6)	11.8 (10.6,13.2)
	3	2763	611	472	344	22.1 (20.6,23.7)	17.1 (15.7,18.5)	12.5 (11.3,13.7)

	4	3049	629	477	337	20.6 (19.2,22.1)	15.6 (14.4,17.0)	11.1 (9.99,12.2)
	5 - least deprived	3504	740	554	396	21.1 (19.8,22.5)	15.8 (14.6,17.1)	11.3 (10.3,12.4)
Current smoker	Current cigarette smoker	1146	238	186	130	20.8 (18.5,23.2)	16.2 (14.2,18.5)	11.3 (9.64,13.3)
	Not current cigarette smoker	11849	2577	1981	1425	21.7 (21.0,22.5)	16.7 (16.1,17.4)	12.0 (11.5,12.6)
	Prefer not to say	175	30	21	17	17.1 (12.3,23.4)	12.0 (7.98,17.6)	9.71 (6.15,15.0)
Current vaper	Current vaper	735	161	123	87	21.9 (19.1,25.0)	16.7 (14.2,19.6)	11.8 (9.70,14.4)
	Not current vaper	12276	2645	2035	1460	21.5 (20.8,22.3)	16.6 (15.9,17.2)	11.9 (11.3,12.5)
	Prefer not to say	159	39	30	25	24.5 (18.5,31.8)	18.9 (13.5,25.7)	15.7 (10.9,22.2)
Severity of COVID-19 symptoms	Mild symptoms	3408	319	224	123	9.36 (8.43,10.4)	6.57 (5.79,7.45)	3.61 (3.03,4.29)
	Moderate symptoms	6703	1325	991	667	19.8 (18.8,20.7)	14.8 (14.0,15.7)	9.95 (9.26,10.7)
	Severe symptoms	3059	1201	973	782	39.3 (37.5,41.0)	31.8 (30.2,33.5)	25.6 (24.0,27.1)
Treatment sought/ received for COVID-19	No medical attention sought	8594	1317	968	605	15.3 (14.6,16.1)	11.3 (10.6,11.9)	7.04 (6.52,7.60)
	Sought medical attention from pharmacist / by phone (NHS 111/GP)	3033	1039	850	680	34.3 (32.6,36.0)	28.0 (26.5,29.7)	22.4 (21.0,23.9)
	Visited GP/walk-in centre/A&E	759	239	174	136	31.5 (28.3,34.9)	22.9 (20.1,26.0)	17.9 (15.4,20.8)
	Hospital admission	168	104	82	71	61.9 (54.4,68.9)	48.8 (41.4,56.3)	42.3 (35.0,49.8)
Gross household income	0-14,999	786	241	186	150	30.7 (27.5,34.0)	23.7 (20.8,26.8)	19.1 (16.5,22.0)
	15,000-49,999	3854	875	692	498	22.7 (21.4,24.1)	18.0 (16.8,19.2)	12.9 (11.9,14.0)
	50,000-149,999	3833	723	535	345	18.9 (17.7,20.1)	14.0 (12.9,15.1)	9.00 (8.14,9.95)
	>150,000	533	69	49	34	12.9 (10.4,16.1)	9.19 (7.02,11.9)	6.38 (4.60,8.78)
Health conditions	Organ transplant recipient	12	3	3	2	25.0 (8.89,53.2)	25.0 (8.89,53.2)	16.7 (4.70,44.8)
	Diabetes (type I or II)	566	167	136	108	29.5 (25.9,33.4)	24.0 (20.7,27.7)	19.1 (16.1,22.5)
	Heart disease or heart problems	416	137	115	95	32.9 (28.6,37.6)	27.6 (23.6,32.1)	22.8 (19.1,27.1)
	Hypertension	1326	395	321	263	29.8 (27.4,32.3)	24.2 (22.0,26.6)	19.8 (17.8,22.1)
	Stroke	73	24	22	17	32.9 (23.2,44.3)	30.1 (20.8,41.4)	23.3 (15.1,34.2)
	Kidney disease	87	28	27	24	32.2 (23.3,42.6)	31.0 (22.3,41.4)	27.6 (19.3,37.8)
	Liver disease	60	24	18	14	40.0 (28.6,52.6)	30.0 (19.9,42.5)	23.3 (14.4,35.4)
	Anaemia	243	75	60	53	30.9 (25.4,36.9)	24.7 (19.7,30.5)	21.8 (17.1,27.4)
	Asthma	1521	478	393	323	31.4 (29.1,33.8)	25.8 (23.7,28.1)	21.2 (19.3,23.4)
	Other lung condition	233	108	90	72	46.4 (40.1,52.8)	38.6 (32.6,45.0)	30.9 (25.3,37.1)
	Cancer	119	27	22	16	22.7 (16.1,31.0)	18.5 (12.5,26.4)	13.4 (8.45,20.7)

Neurological condition	121	38	32	30	31.4 (23.8,40.1)	26.4 (19.4,34.9)	24.8 (18.0,33.2)
Immunocompromised*	477	169	137	114	35.4 (31.3,39.8)	28.7 (24.8,32.9)	23.9 (20.3,27.9)
Depression	1235	404	345	276	32.7 (30.2,35.4)	27.9 (25.5,30.5)	22.3 (20.1,24.8)
Anxiety	1907	576	479	377	30.2 (28.2,32.3)	25.1 (23.2,27.1)	19.8 (18.0,21.6)
Psychiatric disorder (other)	133	47	44	36	35.3 (27.7,43.8)	33.1 (25.7,41.5)	27.1 (20.2,35.2)

Table S6 Round 6 replication of symptom persistence people with symptomatic infections (extended symptom list). Numbers and proportions of participants who reported one or more symptoms (from a list of 37 surveyed symptoms) of COVID-19 at 12 weeks post symptom onset, among the 13,170 symptomatic participants from REACT-2 round 6 for whom we have 12 weeks' observation time and complete data.

			Counts at 12 weeks			*Prevalence at 12 weeks*		
**Variable**	**Category**	**n**	**Number with 1 or more symptoms**	**Number with 2 or more symptoms**	**Number with 3 or more symptoms**	**Prevalence 1 or more symptoms (unweighted)**	**Prevalence 2 or more symptoms (unweighted)**	**Prevalence 3 or more symptoms (unweighted)**
All participants	All participants	13170	3005	2385	1820	22.8 (22.1,23.5)	18.1 (17.5,18.8)	13.8 (13.2,14.4)
Sex	Male	5100	947	716	523	18.6 (17.5,19.7)	14.0 (13.1,15.0)	10.3 (9.45,11.1)
	Female	8069	2058	1669	1297	25.5 (24.6,26.5)	20.7 (19.8,21.6)	16.1 (15.3,16.9)
Age group	18-24	968	116	85	59	12.0 (10.1,14.2)	8.78 (7.16,10.7)	6.10 (4.75,7.78)
	25-34	2266	364	270	180	16.1 (14.6,17.6)	11.9 (10.6,13.3)	7.94 (6.90,9.13)
	35-44	2818	543	432	317	19.3 (17.9,20.8)	15.3 (14.0,16.7)	11.2 (10.1,12.5)
	45-54	3022	757	598	460	25.0 (23.5,26.6)	19.8 (18.4,21.2)	15.2 (14.0,16.5)
	55-64	2520	762	621	492	30.2 (28.5,32.1)	24.6 (23.0,26.4)	19.5 (18.0,21.1)
	65-74	1197	343	277	227	28.7 (26.2,31.3)	23.1 (20.8,25.6)	19.0 (16.8,21.3)
	74+	379	120	102	85	31.7 (27.2,36.5)	26.9 (22.7,31.6)	22.4 (18.5,26.9)
Ethnicity	Asian	590	100	68	53	16.9 (14.1,20.2)	11.5 (9.19,14.4)	8.98 (6.93,11.6)
	Black	204	48	36	28	23.5 (18.2,29.8)	17.6 (13.0,23.5)	13.7 (9.67,19.1)
	Mixed	245	53	44	32	21.6 (16.9,27.2)	18.0 (13.7,23.2)	13.1 (9.41,17.9)
	Other	173	42	30	21	24.3 (18.5,31.2)	17.3 (12.4,23.7)	12.1 (8.08,17.8)
	White	11836	2728	2179	1659	23.0 (22.3,23.8)	18.4 (17.7,19.1)	14.0 (13.4,14.7)
Adiposity	Underweight	163	36	30	20	22.1 (16.4,29.1)	18.4 (13.2,25.1)	12.3 (8.09,18.2)
	Normal weight	4511	880	655	478	19.5 (18.4,20.7)	14.5 (13.5,15.6)	10.6 (9.73,11.5)
	Overweight	4028	871	691	507	21.6 (20.4,22.9)	17.2 (16.0,18.4)	12.6 (11.6,13.6)
	Obese	2816	828	696	570	29.4 (27.7,31.1)	24.7 (23.2,26.3)	20.2 (18.8,21.8)
Healthcare or care home worker	Yes	1342	388	317	233	28.9 (26.5,31.4)	23.6 (21.4,26.0)	17.4 (15.4,19.5)
Index of multiple deprivation (IMD) quintile	1 - most deprived	1480	368	301	241	24.9 (22.7,27.1)	20.3 (18.4,22.5)	16.3 (14.5,18.3)
	2	2374	548	437	338	23.1 (21.4,24.8)	18.4 (16.9,20.0)	14.2 (12.9,15.7)

	3	2763	642	514	394	23.2 (21.7,24.8)	18.6 (17.2,20.1)	14.3 (13.0,15.6)
	4	3049	662	521	394	21.7 (20.3,23.2)	17.1 (15.8,18.5)	12.9 (11.8,14.2)
	5 - least deprived	3504	785	612	453	22.4 (21.1,23.8)	17.5 (16.2,18.8)	12.9 (11.9,14.1)
Current smoker	Current cigarette smoker	1146	251	205	151	21.9 (19.6,24.4)	17.9 (15.8,20.2)	13.2 (11.3,15.3)
	Not current cigarette smoker	11849	2721	2154	1651	23.0 (22.2,23.7)	18.2 (17.5,18.9)	13.9 (13.3,14.6)
	Prefer not to say	175	33	26	18	18.9 (13.8,25.3)	14.9 (10.3,20.9)	10.3 (6.61,15.7)
Current vaper	Current vaper	735	170	133	99	23.1 (20.2,26.3)	18.1 (15.5,21.0)	13.5 (11.2,16.1)
	Not current vaper	12276	2794	2218	1694	22.8 (22.0,23.5)	18.1 (17.4,18.8)	13.8 (13.2,14.4)
	Prefer not to say	159	41	34	27	25.8 (19.6,33.1)	21.4 (15.7,28.4)	17.0 (11.9,23.6)
Severity of COVID-19 symptoms	Mild symptoms	3408	339	236	143	9.95 (8.99,11.0)	6.92 (6.12,7.83)	4.20 (3.57,4.92)
	Moderate symptoms	6703	1404	1082	780	20.9 (20.0,21.9)	16.1 (15.3,17.0)	11.6 (10.9,12.4)
	Severe symptoms	3059	1262	1067	897	41.3 (39.5,43.0)	34.9 (33.2,36.6)	29.3 (27.7,31.0)
Treatment sought/ received for COVID-19	No medical attention sought	8594	1390	1040	711	16.2 (15.4,17.0)	12.1 (11.4,12.8)	8.27 (7.71,8.87)
	Sought medical attention from pharmacist / by phone (NHS 111/GP)	3033	1108	931	782	36.5 (34.8,38.3)	30.7 (29.1,32.4)	25.8 (24.3,27.4)
	Visited GP/walk-in centre/A&E	759	248	198	157	32.7 (29.4,36.1)	26.1 (23.1,29.3)	20.7 (18.0,23.7)
	Hospital admission	168	105	93	79	62.5 (55.0,69.5)	55.4 (47.8,62.7)	47.0 (39.6,54.6)
Gross household income	0-14,999	786	254	208	168	32.3 (29.1,35.7)	26.5 (23.5,29.7)	21.4 (18.7,24.4)
	15,000-49,999	3854	921	740	584	23.9 (22.6,25.3)	19.2 (18.0,20.5)	15.2 (14.1,16.3)
	50,000-149,999	3833	762	586	410	19.9 (18.6,21.2)	15.3 (14.2,16.5)	10.7 (9.76,11.7)
	>150,000	533	75	55	39	14.1 (11.4,17.3)	10.3 (8.01,13.2)	7.32 (5.40,9.85)
Health conditions	Organ transplant recipient	12	3	3	3	25.0 (8.89,53.2)	25.0 (8.89,53.2)	25.0 (8.89,53.2)
	Diabetes (type I or II)	566	176	145	121	31.1 (27.4,35.0)	25.6 (22.2,29.4)	21.4 (18.2,24.9)
	Heart disease or heart problems	416	143	124	105	34.4 (30.0,39.1)	29.8 (25.6,34.4)	25.2 (21.3,29.6)
	Hypertension	1326	418	345	300	31.5 (29.1,34.1)	26.0 (23.7,28.4)	22.6 (20.5,25.0)
	Stroke	73	26	24	20	35.6 (25.6,47.1)	32.9 (23.2,44.3)	27.4 (18.5,38.6)
	Kidney disease	87	28	27	25	32.2 (23.3,42.6)	31.0 (22.3,41.4)	28.7 (20.3,39.0)
	Liver disease	60	25	22	17	41.7 (30.1,54.3)	36.7 (25.6,49.3)	28.3 (18.5,40.8)
	Anaemia	243	83	66	58	34.2 (28.5,40.3)	27.2 (22.0,33.1)	23.9 (18.9,29.6)
	Asthma	1521	495	421	353	32.5 (30.2,34.9)	27.7 (25.5,30.0)	23.2 (21.2,25.4)
	Other lung condition	233	111	98	89	47.6 (41.3,54.0)	42.1 (35.9,48.5)	38.2 (32.2,44.6)

Cancer	119	29	24	19	24.4 (17.5,32.8)	20.2 (13.9,28.3)	16.0 (10.5,23.6)
Neurological condition	121	39	35	31	32.2 (24.6,41.0)	28.9 (21.6,37.6)	25.6 (18.7,34.1)
Immunocompromised*	477	180	148	134	37.7 (33.5,42.2)	31.0 (27.0,35.3)	28.1 (24.2,32.3)
Depression	1235	425	361	313	34.4 (31.8,37.1)	29.2 (26.8,31.8)	25.3 (23.0,27.8)
Anxiety	1907	613	516	431	32.1 (30.1,34.3)	27.1 (25.1,29.1)	22.6 (20.8,24.5)
Psychiatric disorder (other)	133	49	44	39	36.8 (29.1,45.3)	33.1 (25.7,41.5)	29.3 (22.3,37.6)

Table S7 Weighted prevalence of persistent symptoms in the population in round 6.
Numbers and weighted prevalence of currently or previously reported persistent symptoms in the validation REACT-2 study population (rounds 6). Table shows prevalence of any of the 27 surveyed symptoms. The final three columns show prevalence weighted to be representative of the adult population of England, by age, sex, ethnicity, lower-tier local authority population, and index of multiple deprivation (IMD).

			Counts at 12 weeks			*Prevalence at 12 weeks*		
**Variable**	**Category**	**n**	**Number with 1 or more symptoms**	**Number with 2 or more symptoms**	**Number with 3 or more symptoms**	**Prevalence 1 or more symptoms (weighted) %**	**Prevalence 2 or more symptoms (weighted) %**	**Prevalence 3 or more symptoms (weighted) %**
All participants		97727	3094	2384	1729	3.06 (2.98,3.14)	2.28 (2.22,2.36)	1.61 (1.56,1.67)
Sex	Women	56871	2124	1660	1230	2.45 (2.35,2.55)	1.71 (1.63,1.80)	1.13 (1.06,1.20)
	Men	40853	970	724	499	3.64 (3.52,3.76)	2.83 (2.73,2.94)	2.08 (1.99,2.17)
Age group	18-24	4982	115	85	58	2.07 (1.90,2.26)	1.40 (1.26,1.55)	0.977 (0.861,1.11)
	25-34	11791	365	261	163	3.04 (2.87,3.22)	2.07 (1.93,2.22)	1.29 (1.18,1.41)
	35-44	15417	562	437	303	3.63 (3.44,3.82)	2.84 (2.67,3.02)	1.94 (1.80,2.08)
	45-54	18980	771	586	429	4.04 (3.85,4.24)	3.09 (2.92,3.26)	2.32 (2.17,2.47)
	55-64	20760	799	630	470	4.04 (3.63,4.49)	3.23 (2.86,3.64)	2.45 (2.13,2.81)
	65-74	17770	355	284	223	2.02 (1.71,2.39)	1.60 (1.33,1.94)	1.30 (1.05,1.60)
	74+	8027	127	101	83	1.55 (1.40,1.72)	1.25 (1.12,1.40)	0.952 (0.836,1.08)
Ethnicity	Asian	3819	98	69	52	2.22 (1.99,2.47)	1.50 (1.31,1.71)	1.16 (0.998,1.35)
	Black	1214	52	39	30	3.66 (3.19,4.18)	3.11 (2.69,3.60)	2.45 (2.07,2.89)
	Mixed	1224	53	38	32	5.57 (4.72,6.57)	2.85 (2.25,3.59)	2.39 (1.85,3.08)
	Other	968	46	30	20	4.90 (4.07,5.90)	3.01 (2.37,3.81)	2.13 (1.60,2.83)
	White	89720	2811	2179	1571	3.04 (2.95,3.12)	2.30 (2.23,2.38)	1.60 (1.53,1.66)
ADIPOSITY	Normal weight	34950	883	641	446	4.23 (3.56,5.03)	2.67 (2.14,3.33)	2.08 (1.62,2.67)
	Obese	18732	869	709	542	2.49 (2.38,2.62)	1.74 (1.65,1.85)	1.16 (1.08,1.25)
	Overweight	30257	890	689	476	2.87 (2.73,3.02)	2.18 (2.06,2.31)	1.46 (1.36,1.57)
	Underweight	1212	40	27	18	4.41 (4.19,4.64)	3.61 (3.41,3.82)	2.73 (2.55,2.91)
Healthcare or care home worker	Yes	6889	405	308	218	5.23 (4.86,5.62)	4.01 (3.69,4.36)	2.79 (2.52,3.08)
Index of multiple deprivation (IMD) quintile	1 - most deprived	9685	377	307	239	3.41 (3.22,3.60)	2.68 (2.52,2.86)	2.01 (1.87,2.16)
	2	15847	580	456	321	3.29 (3.12,3.48)	2.41 (2.26,2.57)	1.64 (1.52,1.78)
	3	20989	655	504	371	3.01 (2.83,3.19)	2.22 (2.07,2.38)	1.58 (1.46,1.72)

	4	24170	685	516	364	2.69 (2.52,2.87)	1.96 (1.82,2.11)	1.33 (1.21,1.46)
	5 - least deprived	27036	797	601	434	2.83 (2.66,3.02)	2.12 (1.97,2.28)	1.47 (1.35,1.61)
Current smoker	Current cigarette smoker	8041	266	206	148	3.26 (3.01,3.53)	2.50 (2.28,2.74)	1.57 (1.39,1.76)
	Not current cigarette smoker	88392	2791	2153	1561	3.05 (2.96,3.13)	2.27 (2.20,2.35)	1.63 (1.57,1.69)
	Prefer not to say	1294	37	25	20	2.26 (1.77,2.87)	1.55 (1.16,2.08)	1.09 (0.772,1.55)
Current vaper	Current vaper	4942	175	134	97	3.48 (3.16,3.83)	2.70 (2.42,3.02)	1.86 (1.63,2.12)
	Not current vaper	91627	2877	2219	1606	3.03 (2.95,3.11)	2.26 (2.19,2.33)	1.60 (1.54,1.66)
	Prefer not to say	1158	42	31	26	2.87 (2.28,3.61)	2.13 (1.63,2.79)	1.68 (1.24,2.27)
Severity of COVID symptoms	Mild symptoms	3817	338	235	132	7.48 (6.96,8.03)	4.70 (4.29,5.15)	2.54 (2.24,2.88)
	Moderate symptoms	7367	1425	1070	723	16.1 (15.6,16.7)	11.9 (11.4,12.5)	7.88 (7.47,8.32)
	Severe symptoms	3378	1319	1071	866	32.3 (31.2,33.4)	25.7 (24.6,26.7)	20.4 (19.4,21.4)
Treatment sought/ received for COVID	No medical attention sought	198	117	93	80	12.4 (11.9,12.8)	8.75 (8.37,9.14)	5.25 (4.96,5.56)
	Sought medical attention from pharmacist / by phone (NHS 111/GP)	9450	1407	1037	657	27.0 (26.0,28.0)	21.4 (20.5,22.4)	17.4 (16.5,18.3)
	Visited GP/walk-in centre/A&E	3418	1138	931	742	25.2 (23.0,27.4)	19.0 (17.1,21.1)	14.8 (13.1,16.7)
	Hospital admission	817	259	191	152	52.7 (47.7,57.7)	42.3 (37.5,47.3)	33.8 (29.2,38.6)
Gross household income	0-14,999	2906	73	53	37	3.71 (3.40,4.05)	2.72 (2.45,3.01)	2.24 (2.00,2.51)
	15,000-49,999	6882	277	214	172	3.30 (3.15,3.46)	2.50 (2.37,2.64)	1.75 (1.64,1.87)
	50,000-149,999	29163	966	764	558	3.04 (2.89,3.20)	2.26 (2.13,2.41)	1.42 (1.32,1.54)
	>150,000	23678	759	565	366	2.29 (1.91,2.73)	1.63 (1.32,2.01)	1.12 (0.870,1.45)
Comorbidities	Organ transplant recipient	115	3	3	2	3.89 (1.90,7.81)	3.89 (1.90,7.81)	1.11 (0.305,3.96)
	Diabetes (type I or II)	5537	187	153	122	3.38 (3.00,3.80)	2.84 (2.50,3.23)	2.24 (1.93,2.59)
	Heart disease or heart problems	4170	158	134	113	3.16 (2.75,3.62)	2.78 (2.40,3.22)	2.23 (1.89,2.63)

Hypertension (high blood pressure)	12958	436	357	293	3.19 (2.93,3.46)	2.58 (2.35,2.83)	2.14 (1.93,2.37)
Stroke	591	24	22	17	1.93 (1.21,3.07)	1.82 (1.12,2.93)	1.48 (0.864,2.51)
Kidney disease	750	33	31	28	5.70 (4.50,7.19)	4.92 (3.82,6.32)	4.15 (3.14,5.45)
Liver disease	426	29	23	18	8.24 (6.41,10.5)	6.37 (4.78,8.44)	4.63 (3.30,6.46)
Anaemia	1532	95	80	71	6.13 (5.37,6.99)	4.86 (4.18,5.63)	4.53 (3.88,5.29)
Asthma	9508	518	426	352	4.65 (4.36,4.97)	3.88 (3.61,4.17)	3.23 (2.98,3.50)
Other lung condition	2333	117	96	77	4.72 (4.05,5.50)	3.60 (3.01,4.29)	3.08 (2.54,3.73)
Cancer	1626	34	27	21	2.10 (1.58,2.80)	1.78 (1.30,2.43)	1.54 (1.10,2.16)
Condition affecting the brain and nerves	1120	41	34	32	3.44 (2.71,4.35)	2.55 (1.93,3.36)	2.45 (1.84,3.24)
A weakened immune system/reduced ability to deal with infections	3662	190	154	130	5.64 (5.07,6.27)	4.73 (4.21,5.31)	4.02 (3.54,4.57)
Depression	7262	445	382	308	6.03 (5.66,6.41)	4.96 (4.63,5.31)	3.93 (3.64,4.25)
Anxiety	10687	633	526	417	5.40 (5.11,5.70)	4.53 (4.27,4.81)	3.51 (3.28,3.75)
Psychiatric disorder	634	52	48	40	8.80 (7.50,10.3)	7.98 (6.74,9.42)	5.70 (4.66,6.95)

Table S8 Weighted prevalence of persistent symptoms in the population in round 6 (extended symptom set). Numbers and weighted prevalence of currently or previously reported persistent symptoms in the validation REACT-2 study population (rounds 6). Table shows prevalence of any of the 37 surveyed symptoms. The final three columns show prevalence weighted to be representative of the adult population of England, by age, sex, ethnicity, lower-tier local authority population, and index of multiple deprivation (IMD).

			Counts at 12 weeks			*Prevalence at 12 weeks*		
**Variable**	**Category**	**n**	**Number with 1 or more symptoms**	**Number with 2 or more symptoms**	**Number with 3 or more symptoms**	**Prevalence 1 or more symptoms (weighted) %**	**Prevalence 2 or more symptoms (weighted) %**	**Prevalence 3 or more symptoms (weighted) %**
All participants		97727	3274	2606	2001	3.26 (3.18,3.34)	2.50 (2.43,2.57)	1.86 (1.80,1.92)
Sex	Women	40853	1032	784	577	2.63 (2.53,2.74)	1.91 (1.82,2.00)	1.32 (1.24,1.40)
	Men	56871	2242	1822	1424	3.86 (3.74,3.99)	3.07 (2.96,3.18)	2.38 (2.28,2.48)
Age group	18-24	4982	121	90	64	2.21 (2.03,2.40)	1.52 (1.37,1.68)	1.04 (0.923,1.18)
	25-34	11791	384	286	191	3.25 (3.08,3.43)	2.31 (2.16,2.46)	1.55 (1.43,1.68)
	35-44	15417	601	483	357	3.96 (3.76,4.16)	3.12 (2.95,3.31)	2.31 (2.16,2.47)
	45-54	18980	811	641	492	4.24 (4.05,4.45)	3.34 (3.17,3.53)	2.60 (2.45,2.76)
	55-64	20760	845	691	553	4.31 (3.89,4.78)	3.53 (3.14,3.95)	2.80 (2.46,3.19)
	65-74	17770	380	305	252	2.18 (1.85,2.56)	1.71 (1.42,2.06)	1.45 (1.19,1.77)
	74+	8027	132	110	92	1.61 (1.46,1.78)	1.35 (1.21,1.50)	1.10 (0.971,1.24)
Ethnicity	Asian	3819	113	78	60	2.65 (2.39,2.92)	1.68 (1.48,1.91)	1.32 (1.14,1.52)
	Black	1214	58	43	35	4.07 (3.58,4.62)	3.22 (2.79,3.72)	2.70 (2.31,3.16)
	Mixed	1224	56	47	35	5.99 (5.11,7.02)	3.52 (2.85,4.33)	2.60 (2.03,3.31)
	Other	968	50	37	24	5.18 (4.32,6.20)	4.12 (3.36,5.04)	2.45 (1.88,3.19)
	White	89720	2961	2371	1818	3.21 (3.12,3.30)	2.50 (2.42,2.58)	1.85 (1.78,1.92)
ADIPOSITY	Underweight	1212	40	34	24	4.23 (3.56,5.03)	3.23 (2.64,3.94)	2.53 (2.02,3.17)
	Normal weight	34950	944	708	519	2.66 (2.54,2.79)	1.94 (1.83,2.05)	1.34 (1.25,1.43)
	Overweight	30257	951	754	554	3.10 (2.96,3.26)	2.37 (2.24,2.50)	1.70 (1.60,1.82)
	Obese	18732	905	759	627	4.66 (4.43,4.89)	3.89 (3.68,4.11)	3.09 (2.91,3.29)
Healthcare or care home worker	Yes	6889	430	351	263	5.61 (5.23,6.02)	4.44 (4.10,4.80)	3.29 (3.00,3.61)
Index of multiple deprivation (IMD)	1 - most deprived	9685	404	332	267	3.72 (3.53,3.92)	2.88 (2.71,3.06)	2.25 (2.11,2.41)
	2	15847	610	489	383	3.52 (3.34,3.71)	2.65 (2.49,2.82)	1.92 (1.79,2.06)

quintile	3	20989	692	552	425	3.14 (2.96,3.32)	2.42 (2.27,2.58)	1.86 (1.72,2.00)
	4	24170	722	568	431	2.86 (2.69,3.04)	2.14 (1.99,2.30)	1.57 (1.45,1.71)
	5 - least deprived	27036	846	665	495	3.00 (2.82,3.19)	2.37 (2.21,2.54)	1.66 (1.53,1.80)
Current smoker	Current cigarette smoker	8041	280	227	170	3.48 (3.23,3.76)	2.75 (2.52,3.00)	1.98 (1.79,2.19)
	Not current cigarette smoker	88392	2954	2347	1809	3.25 (3.16,3.34)	2.48 (2.40,2.56)	1.86 (1.79,1.93)
	Prefer not to say	1294	40	32	22	2.51 (1.99,3.15)	2.08 (1.62,2.68)	1.13 (0.801,1.59)
Current vaper	Current vaper	4942	185	146	111	3.87 (3.53,4.24)	2.90 (2.61,3.23)	2.04 (1.80,2.32)
	Not current vaper	91627	3045	2424	1862	3.22 (3.14,3.31)	2.47 (2.40,2.55)	1.85 (1.78,1.91)
	Prefer not to say	1158	44	36	28	2.95 (2.35,3.70)	2.46 (1.92,3.16)	1.76 (1.31,2.37)
Severity of COVID symptoms	Mild symptoms	3817	360	247	153	8.00 (7.47,8.57)	4.88 (4.46,5.34)	2.95 (2.62,3.31)
	Moderate symptoms	7367	1510	1169	850	17.2 (16.6,17.8)	13.0 (12.4,13.5)	9.20 (8.75,9.67)
	Severe symptoms	3378	1390	1178	990	34.3 (33.2,35.5)	28.6 (27.6,29.7)	23.2 (22.2,24.2)
Treatment sought/ received for COVID	No medical attention sought	9450	1485	1114	771	13.1 (12.6,13.5)	9.38 (8.99,9.78)	6.23 (5.91,6.57)
	Sought medical attention from pharmacist / by phone (NHS 111/GP)	3418	1215	1023	857	29.4 (28.3,30.4)	23.6 (22.7,24.6)	19.8 (18.9,20.7)
	Visited GP/walk-in centre/A&E	817	271	216	173	26.7 (24.5,29.0)	21.5 (19.5,23.7)	16.1 (14.3,18.0)
	Hospital admission	198	120	106	91	53.9 (48.9,58.8)	49.7 (44.8,54.7)	38.4 (33.7,43.4)
Gross household income	0-14,999	6882	293	239	193	3.87 (3.55,4.21)	3.17 (2.88,3.49)	2.54 (2.29,2.83)
	15,000-49,999	29163	1019	822	652	3.59 (3.43,3.75)	2.68 (2.54,2.82)	2.04 (1.92,2.16)
	50,000-149,999	23678	801	617	435	3.21 (3.06,3.38)	2.47 (2.33,2.61)	1.67 (1.56,1.80)
	>150,000	2906	79	59	42	2.52 (2.13,2.98)	1.84 (1.51,2.24)	1.24 (0.972,1.58)
Comorbidities	Organ transplant recipient	115	3	3	3	3.89 (1.90,7.81)	3.89 (1.90,7.81)	3.89 (1.90,7.81)
	Diabetes (type I or II)	5537	197	164	138	3.56 (3.18,4.00)	3.06 (2.70,3.46)	2.54 (2.22,2.91)
	Heart disease or heart problems	4170	164	144	124	3.22 (2.81,3.69)	3.01 (2.61,3.47)	2.51 (2.15,2.93)
	Hypertension (high blood pressure)	12958	461	386	336	3.39 (3.13,3.68)	2.81 (2.57,3.07)	2.40 (2.18,2.65)
	Stroke	591	26	24	20	2.15 (1.38,3.34)	1.93 (1.21,3.07)	1.82 (1.12,2.93)
	Kidney disease	750	33	31	29	5.70 (4.50,7.19)	4.92 (3.82,6.32)	4.49 (3.44,5.84)
	Liver disease	426	30	27	22	8.24 (6.41,10.5)	7.95 (6.16,10.2)	6.37 (4.78,8.44)
	Anaemia	1532	103	86	76	6.99 (6.18,7.90)	5.27 (4.57,6.08)	4.59 (3.93,5.35)
	Asthma	9508	537	458	384	4.75 (4.45,5.08)	4.13 (3.85,4.43)	3.48 (3.22,3.76)
	Other lung condition	2333	120	106	94	4.82 (4.14,5.60)	4.18 (3.54,4.92)	3.63 (3.04,4.32)

Cancer	1626	36	29	24	2.15 (1.62,2.86)	1.87 (1.38,2.54)	1.54 (1.10,2.16)
Condition affecting the brain and nerves	1120	42	37	33	3.64 (2.89,4.58)	3.02 (2.34,3.88)	2.60 (1.98,3.41)
A weakened immune system/reduced ability to deal with infections	3662	201	167	152	5.94 (5.36,6.58)	4.94 (4.41,5.53)	4.60 (4.09,5.18)
Depression	7262	466	400	348	6.36 (5.99,6.75)	5.26 (4.92,5.63)	4.41 (4.10,4.75)
Anxiety	10687	670	569	475	5.83 (5.53,6.14)	4.87 (4.60,5.15)	3.92 (3.67,4.18)
Psychiatric disorder	634	54	49	43	8.92 (7.62,10.4)	8.04 (6.80,9.49)	6.77 (5.64,8.12)

Table S9 Odds ratios for persistent symptoms at 12 weeks among symptomatic respondents.

Derived from logistic regression models (forest plot shown in Figure 2)

Predictor	Level	Crude (univariable) OR	Adjusted on age and sex	Mutually adjusted*
Sex	Male [reference]	-	-	-
	Female	1.46 [1.42-1.51]	1.51 [1.46-1.55]	1.38 [1.32-1.45]
Age group	18-24	0.58 [0.54-0.62]	0.56 [0.53-0.6]	0.64 [0.57-0.72]
	25-34	0.6 [0.57-0.63]	0.58 [0.55-0.61]	0.6 [0.56-0.65]
	35-44	0.65 [0.62-0.68]	0.64 [0.61-0.67]	0.64 [0.59-0.68]
	45-54	0.86 [0.83-0.9]	0.86 [0.82-0.9]	0.85 [0.8-0.91]
	55-64 [reference]	-	-	-
	65-74	1.16 [1.1-1.22]	1.16 [1.1-1.23]	1.16 [1.01-1.33]
	74+	1.51 [1.38-1.64]	1.47 [1.35-1.61]	1.19 [0.71-1.98]
Ethnicity	White [reference]	-	-	-
	Asian	0.71 [0.65-0.77]	0.8 [0.74-0.88]	0.84 [0.74-0.96]
	Black	0.99 [0.85-1.15]	1.06 [0.91-1.24]	1.02 [0.81-1.28]
	Mixed	1.05 [0.94-1.18]	1.19 [1.06-1.33]	1.16 [0.98-1.38]
	Other	0.99 [0.86-1.14]	1.05 [0.91-1.22]	1.05 [0.83-1.32]
Adiposity	Underweight	1.15 [1.01-1.33]	1.17 [1.02-1.35]	0.99 [0.79-1.25]
	Normal weight [reference]	-	-	-
	Overweight	1.14 [1.1-1.18]	1.16 [1.12-1.21]	1.15 [1.09-1.21]
	Obese	1.52 [1.46-1.58]	1.53 [1.47-1.59]	1.39 [1.32-1.48]
Healthcare or care home worker	No [reference]	-	-	-
	Yes	1.48 [1.39-1.56]	1.33 [1.26-1.41]	1.26 [1.17-1.36]
Index of multiple deprivation (IMD) quintile	1 - most deprived	1.19 [1.12-1.25]	1.24 [1.17-1.31]	1.09 [1-1.19]
	2	1.05 [1-1.1]	1.08 [1.03-1.13]	1.04 [0.97-1.12]
	3 [reference]	-	-	-
	4	0.94 [0.9-0.98]	0.92 [0.88-0.97]	0.98 [0.92-1.05]
	5 - least deprived	0.85 [0.82-0.89]	0.84 [0.8-0.87]	0.91 [0.85-0.97]
Current smoker	Not current cigarette smoker [reference]	-	-	-
	Current cigarette smoker	1.23 [1.17-1.29]	1.35 [1.28-1.41]	1.11 [1.03-1.2]
	Prefer not to say	0.92 [0.79-1.08]	1.03 [0.88-1.21]	0.84 [0.63-1.14]
Current vaper	Not current vaper [reference]	-	-	-
	Current vaper	1.12 [1.06-1.19]	1.26 [1.18-1.34]	1.11 [1.01-1.21]
	Prefer not to say	0.84 [0.69-1.03]	0.94 [0.77-1.16]	0.94 [0.59-1.51]
Severity of COVID-19 symptoms	Mild symptoms [reference]	-	-	-
	Moderate symptoms	1.82 [1.75-1.89]	1.74 [1.68-1.81]	1.51 [1.43-1.6]
	Severe symptoms	3.25 [3.11-3.39]	3.02 [2.89-3.15]	2.26 [2.1-2.42]
Treatment sought/ received for COVID-19	No medical attention sought [reference]	-	-	-
	Pharmacist / by phone (NHS 111/GP)	2.07 [2-2.14]	2.05 [1.98-2.12]	2.02 [1.92-2.13]
	Visited GP/walk-in centre/A&E	1.81 [1.72-1.91]	1.67 [1.58-1.76]	1.57 [1.44-1.71]
	Hospital admission	3.98 [3.37-4.69]	3.46 [2.93-4.09]	3.45 [2.57-4.64]
Gross household income	0-14,999	1.51 [1.42-1.61]	1.39 [1.3-1.48]	1.36 [1.22-1.51]
	15,000-49,999 [reference]	-	-	-
	50,000-149,999	0.7 [0.67-0.72]	0.76 [0.73-0.79]	0.84 [0.8-0.88]

	>150,000	0.59 [0.54-0.64]	0.64 [0.58-0.69]	0.8 [0.73-0.88]
Hospitalised with COVID	No [reference]	-	-	-
	Yes	3.08 [2.61-3.63]	2.72 [2.3-3.21]	3.45 [2.57-4.64]

**Owing to missing data in some variables, the total n for the mutually adjusted model is 55,730*

Table S10 Characteristics of 12-week symptom clusters in rounds 3–5.

Characteristics of 12-week symptom clusters among 70% sub-sample (n=53,309) of people with symptoms and date of symptom onset 12 weeks or more before survey date -- compared with those who were not experiencing any persistent symptoms at 12 weeks. For categorical variables, column-wise within-group percentages shown in parentheses, with 95% confidence intervals in square brackets. Cluster L2 (respiratory) contained proportionately more people who were obese (31.6%, vs 27.0% in Cluster L1), more smokers (12.9% vs 12.2%) and more people who rated their symptoms as 'severe' (43.5% vs 27.4%). Prevalence of all surveyed comorbidities was higher in Cluster L2 than Cluster L1, and the prevalence of comorbidities was higher in the symptomatic clusters than in those who did not experience persistent symptoms at 12 weeks.

Variable	Category	Asymptomatic at 12 weeks	Cluster L1 ("tiredness")	Cluster L2 ("respiratory")	Total /mean (SD)
All participants	All participants	33069 (100%, [100-100])	15799 (100%, [100-100])	4441 (100%, [99.9-100])	53309
Sex	Women	17834 (53.9%, [53.4-54.5])	10041 (63.6%, [62.8-64.3])	2794 (62.9%, [61.5-64.3])	30669
	Men	15234 (46.1%, [45.5-46.6])	5758 (36.4%, [35.7-37.2])	1647 (37.1%, [35.7-38.5])	22639
Age group	18-24	2440 (7.4%, [7.1-7.7])	917 (5.8%, [5.5-6.2])	185 (4.2%, [3.6-4.8])	3542
	25-34	5911 (17.9%, [17.5-18.3])	2108 (13.3%, [12.8-13.9])	547 (12.3%, [11.4-13.3])	8566
	35-44	7478 (22.6%, [22.2-23.1])	2891 (18.3%, [17.7-18.9])	836 (18.8%, [17.7-20])	11205
	45-54	7476 (22.6%, [22.2-23.1])	3687 (23.3%, [22.7-24])	1108 (24.9%, [23.7-26.2])	12271
	55-64	6021 (18.2%, [17.8-18.6])	3492 (22.1%, [21.5-22.8])	1035 (23.3%, [22.1-24.6])	10548
	65-74	2971 (9%, [8.7-9.3])	2020 (12.8%, [12.3-13.3])	544 (12.2%, [11.3-13.2])	5535
	74+	772 (2.3%, [2.2-2.5])	684 (4.3%, [4-4.7])	186 (4.2%, [3.6-4.8])	1642
Ethnicity	Asian	1337 (4.1%, [3.9-4.3])	464 (3%, [2.7-3.2])	98 (2.2%, [1.8-2.7])	1899
	Black	319 (1%, [0.9-1.1])	168 (1.1%, [0.9-1.2])	34 (0.8%, [0.6-1.1])	521
	Mixed	524 (1.6%, [1.5-1.7])	262 (1.7%, [1.5-1.9])	92 (2.1%, [1.7-2.6])	878
	Other	354 (1.1%, [1-1.2])	164 (1%, [0.9-1.2])	42 (1%, [0.7-1.3])	560
	White	30275 (92.3%, [92-92.6])	14614 (93.2%, [92.8-93.6])	4146 (94%, [93.2-94.6])	49035
ADIPOSITY	Normal weight	11981 (41.2%, [40.6-41.8])	5071 (36.5%, [35.7-37.3])	1241 (31.8%, [30.4-33.3])	18293
	Obese	6245 (21.5%, [21-21.9])	3756 (27%, [26.3-27.8])	1234 (31.6%, [30.2-33.1])	11235
	Overweight	10476 (36%, [35.5-36.6])	4898 (35.3%, [34.5-36.1])	1365 (35%, [33.5-36.5])	16739
	Underweight	381 (1.3%, [1.2-1.4])	166 (1.2%, [1-1.4])	63 (1.6%, [1.3-2.1])	610
Healthcare or care home worker	Yes	2109 (8.5%, [8.2-8.9])	1260 (11.8%, [11.2-12.4])	358 (12.2%, [11.1-13.4])	3727
Index of multiple deprivation (IMD) quintile	1 - most deprived	3244 (9.8%, [9.5-10.1])	1845 (11.7%, [11.2-12.2])	586 (13.2%, [12.2-14.2])	5675
	2	5463 (16.5%, [16.1-16.9])	2739 (17.3%, [16.8-17.9])	794 (17.9%, [16.8-19])	8996
	3	7123 (21.5%, [21.1-22])	3504 (22.2%, [21.5-22.8])	1008 (22.7%, [21.5-24])	11635

	4	8004 (24.2%, [23.7-24.7])	3790 (24%, [23.3-24.7])	1000 (22.5%, [21.3-23.8])	12794
	5 - least deprived	9235 (27.9%, [27.4-28.4])	3921 (24.8%, [24.2-25.5])	1053 (23.7%, [22.5-25])	14209
Current smoker	Current cigarette smoker	3430 (10.4%, [10-10.7])	1935 (12.2%, [11.7-12.8])	573 (12.9%, [11.9-13.9])	5938
	Not current cigarette smoker	29321 (88.7%, [88.3-89])	13731 (86.9%, [86.4-87.4])	3826 (86.2%, [85.1-87.1])	46878
	Prefer not to say	318 (1%, [0.9-1.1])	133 (0.8%, [0.7-1])	42 (0.9%, [0.7-1.3])	493
Current vaper	Current vaper	2042 (6.2%, [5.9-6.4])	1083 (6.9%, [6.5-7.3])	328 (7.4%, [6.7-8.2])	3453
	Not current vaper	30831 (93.2%, [93-93.5])	14639 (92.7%, [92.2-93.1])	4086 (92%, [91.2-92.8])	49556
	Prefer not to say	196 (0.6%, [0.5-0.7])	77 (0.5%, [0.4-0.6])	27 (0.6%, [0.4-0.9])	300
Severity of COVID symptoms	Mild symptoms	11133 (33.7%, [33.2-34.2])	3345 (21.2%, [20.5-21.8])	476 (10.7%, [9.8-11.7])	14954
	Moderate symptoms	16378 (49.5%, [49-50.1])	8130 (51.5%, [50.7-52.2])	2034 (45.8%, [44.3-47.3])	26542
	Severe symptoms	5558 (16.8%, [16.4-17.2])	4324 (27.4%, [26.7-28.1])	1931 (43.5%, [42.0-44.9])	11813
Treatment sought/ received for COVID	Hospital admission	149 (0.5%, [0.4-0.6])	163 (1.1%, [0.9-1.3])	122 (2.9%, [2.5-3.5])	434
	No medical attention sought	22687 (72%, [71.5-72.5])	8811 (59.2%, [58.4-59.9])	1758 (42.3%, [40.8-43.8])	33256
	Sought medical attention from pharmacist / by phone (NHS 111/GP)	6355 (20.2%, [19.7-20.6])	4525 (30.4%, [29.6-31.1])	1674 (40.3%, [38.8-41.7])	12554
	Visited GP/walk-in centre/A&E	2324 (7.4%, [7.1-7.7])	1397 (9.4%, [8.9-9.9])	605 (14.5%, [13.5-15.7])	4326
Gross household income	>150,000	1516 (6.8%, [6.5-7.2])	509 (4.9%, [4.5-5.3])	109 (3.7%, [3.1-4.4])	2134
	0-14,999	1545 (7%, [6.6-7.3])	1222 (11.7%, [11.1-12.3])	405 (13.8%, [12.6-15.1])	3172
	15,000-49,999	8858 (39.9%, [39.2-40.5])	4743 (45.4%, [44.5-46.4])	1380 (46.9%, [45.1-48.7])	14981
	50,000-149,999	10296 (46.3%, [45.7-47])	3968 (38%, [37.1-38.9])	1049 (35.6%, [33.9-37.4])	15313
Comorbidities	Organ transplant recipient	26 (0.1%, [0.1-0.1])	14 (0.1%, [0.1-0.1])	9 (0.2%, [0.1-0.4])	49
	Diabetes (type I or II)	1171 (3.5%, [3.3-3.7])	843 (5.3%, [5-5.7])	258 (5.8%, [5.2-6.5])	2272
	Heart disease or heart problems	914 (2.8%, [2.6-2.9])	777 (4.9%, [4.6-5.3])	288 (6.5%, [5.8-7.2])	1979
	Hypertension (high blood pressure)	3004 (9.1%, [8.8-9.4])	2165 (13.7%, [13.2-14.2])	644 (14.5%, [13.5-15.6])	5813
	Kidney disease	146 (0.4%, [0.4-0.5])	106 (0.7%, [0.6-0.8])	47 (1.1%, [0.8-1.4])	299
	Liver disease	158 (0.5%, [0.4-0.6])	159 (1%, [0.9-1.2])	54 (1.2%, [0.9-1.6])	371
	Anaemia	87 (0.3%, [0.2-0.3])	84 (0.5%, [0.4-0.7])	32 (0.7%, [0.5-1])	203
	Asthma	381 (1.2%, [1-1.3])	432 (2.7%, [2.5-3])	145 (3.3%, [2.8-3.8])	958
	Other lung condition	3264 (9.9%, [9.6-10.2])	2001 (12.7%, [12.2-13.2])	1113 (25.1%, [23.8-26.4])	6378
	Cancer	347 (1%, [0.9-1.2])	435 (2.8%, [2.5-3])	362 (8.2%, [7.4-9])	1144
	Condition affecting the brain and nerves	284 (0.9%, [0.8-1])	208 (1.3%, [1.2-1.5])	65 (1.5%, [1.2-1.9])	557
	A weakened immune system/reduced ability to deal with infections	220 (0.7%, [0.6-0.8])	211 (1.3%, [1.2-1.5])	65 (1.5%, [1.2-1.9])	496

Depression	876 (2.6%, [2.5-2.8])	917 (5.8%, [5.5-6.2])	364 (8.2%, [7.4-9])	2157
Anxiety	2139 (6.5%, [6.2-6.7])	2057 (13%, [12.5-13.6])	695 (15.6%, [14.6-16.7])	4891
Psychiatric disorder	3504 (10.6%, [10.3-10.9])	2940 (18.6%, [18-19.2])	998 (22.5%, [21.3-23.7])	7442
None of these	189 (0.6%, [0.5-0.7])	188 (1.2%, [1-1.4])	76 (1.7%, [1.4-2.1])	453

Table S11 Summary of persistent symptom prevalence among LFIA-positive individuals.
Numbers and proportions of participants who reported one or more symptoms (from a list of 29 surveyed symptoms) of COVID-19 at i) time of symptom onset and ii) 12 weeks post symptom onset, among a subset of 14,704 symptomatic participants who tested positive for antibodies by lateral flow immunoassay, and for whom we have 12 weeks' observation time and complete data.

Variable	Category	Symptomatic at infection	Number symptomatic at 12 weeks	% symptomatic at 12 weeks
All participants	-	14704	6232	42.4%, [41.6-43.2]
Sex	Female	8546	4022	47.1%, [46-48.1]
	Male	6158	2210	35.9%, [34.7-37.1]
Age group	18-24	1210	384	31.7%, [29.2-34.4]
	25-34	2410	841	34.9%, [33-36.8]
	35-44	2615	1011	38.7%, [36.8-40.5]
	45-54	3419	1552	45.4%, [43.7-47.1]
	55-64	3180	1502	47.2%, [45.5-49]
	65-74	1395	691	49.5%, [46.9-52.2]
	74+	475	251	52.8%, [48.3-57.3]
Ethnicity	Asian	877	301	34.3%, [31.3-37.5]
	Black	215	93	43.3%, [36.8-49.9]
	Mixed	241	94	39%, [33.1-45.3]
	Other	223	87	39%, [32.8-45.5]
	White	13024	5606	43%, [42.2-43.9]

Table S12 Symptom prevalence in PCR-positive individuals in REACT-1.

Proportion of PCR-positive respondents reporting one or more of 26 symptoms up to 7 days before the PCR test, among 7,204 total PCR-positive adults (18+) surveyed in rounds 2 to 14 of REACT-1, between 20 June 2020 and 27 September 2021. A decline in the proportion of respondents reporting symptoms is observed in participants in the oldest age groups.

Variable	Category	Total PCR positive	Asymptomatic	Symptomatic
Age	18-24	596	344 (57.7%, [53.7-61.6])	252 (42.3%, [38.4-46.3])
Age	25-34	928	484 (52.2%, [48.9-55.4])	444 (47.8%, [44.6-51.1])
Age	35-44	1196	636 (53.2%, [50.3-56])	560 (46.8%, [44-49.7])
Age	45-54	1556	816 (52.4%, [50-54.9])	740 (47.6%, [45.1-50])
Age	55-64	1480	825 (55.7%, [53.2-58.3])	655 (44.3%, [41.7-46.8])
Age	65+	1448	1007 (69.5%, [67.1-71.9])	441 (30.5%, [28.1-32.9])

Figure S1 Modelled probability of symptomatic infection and persistent symptoms, by age.
Plots showing (top row) modelled probability of persistent symptoms at 12 weeks as a function of age and sex, using generalised additive models with splines on age and interactions between age and sex (top row), and (bottom row) weighted prevalence of persistent symptoms at 12 weeks. From left to right, plots show (i) probability of experiencing COVID-19 symptoms in the population (n=508,707 observations), (ii) probability of experiencing persistent symptoms at 12 weeks in the population, and (iii) probability of experiencing persistent symptoms at 12 weeks, conditional on symptomatic infection (n=71,642 observations for whom we had 150 days' observation time). Conditional on symptomatic infection (likelihood of which decreases after age 50, see central panels), risk of persistent symptoms increases linearly with age (right panels).

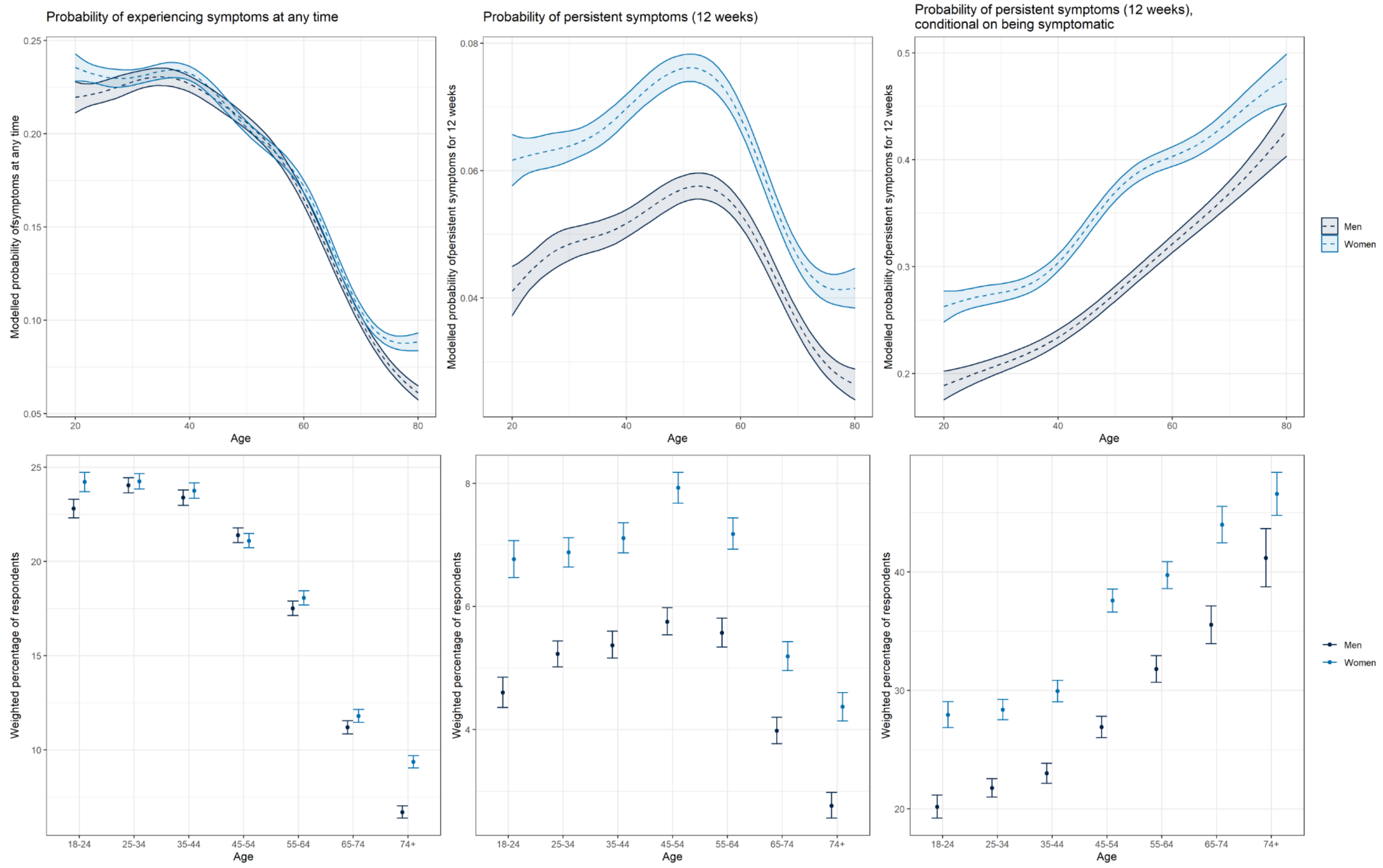

Figure S2 Modelling of persistent symptoms as a function of biological and demographic variables (round 6 replication).

Results of replication analysis in round 6 of REACT2 (data collected between 12 and 25 May, 2021. N=13,170 observations, all self-reporting previous symptomatic COVID-19). (A) Logistic regression models with one or more symptoms at 12 weeks (y/n) as the binary outcome variable, both unadjusted and adjusted for sex and age; (B) Mean contribution to area under the curve (AUC) that each variable makes to a multivariable boosted tree model; (C) modelled probability of persistent symptoms at 12 weeks as a function of age and sex, using generalised additive models with splines on age and interactions between age and sex. The results are consistent with results from the main study population (Figure 2): Age, sex, adiposity and prior hospitalisation with COVID-19 are the strongest predictors of persistent symptoms in multivariable modelling, while Asian ethnicity is associated with a lower risk of persistent symptoms at 12 weeks. Current smoker status, household income, healthcare/care home worker status and deprivation were not selected in multivariable variable selection in round 6 but were selected in the main analysis in rounds 3–5 (Figure 2). Box plots in panel B show median, first and third quartiles; whiskers indicate 1.5 * the interquartile range; data beyond this range are plotted as points.

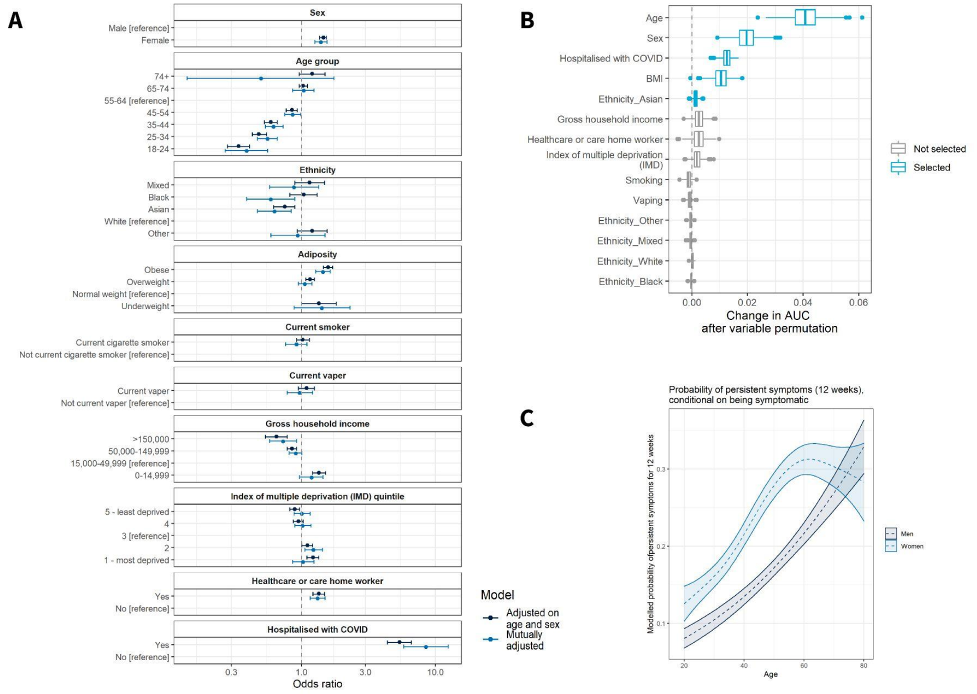

Figure S3 Silhouette plot showing average silhouette width for 2 to 10 clusters.
Clustering on symptoms at 12 weeks after initial symptom onset. The optimal number of clusters by this metric was 2.

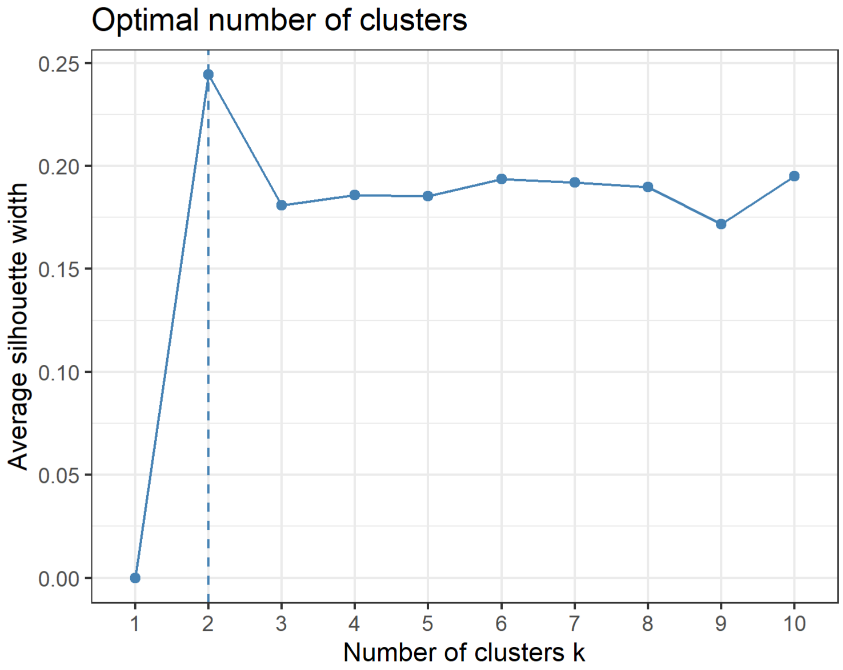

Figure S4 Pairwise co-occurrence heatmaps of symptoms at 12 weeks after symptom onset, by cluster. Cluster L1 (left) and Cluster L2 (right), in rounds 3–5. Deeper blue colour indicates that more people had both paired symptoms at 12 weeks post symptom onset; bar plots indicate marginal counts of participants with each symptom at 12 weeks post symptom onset.

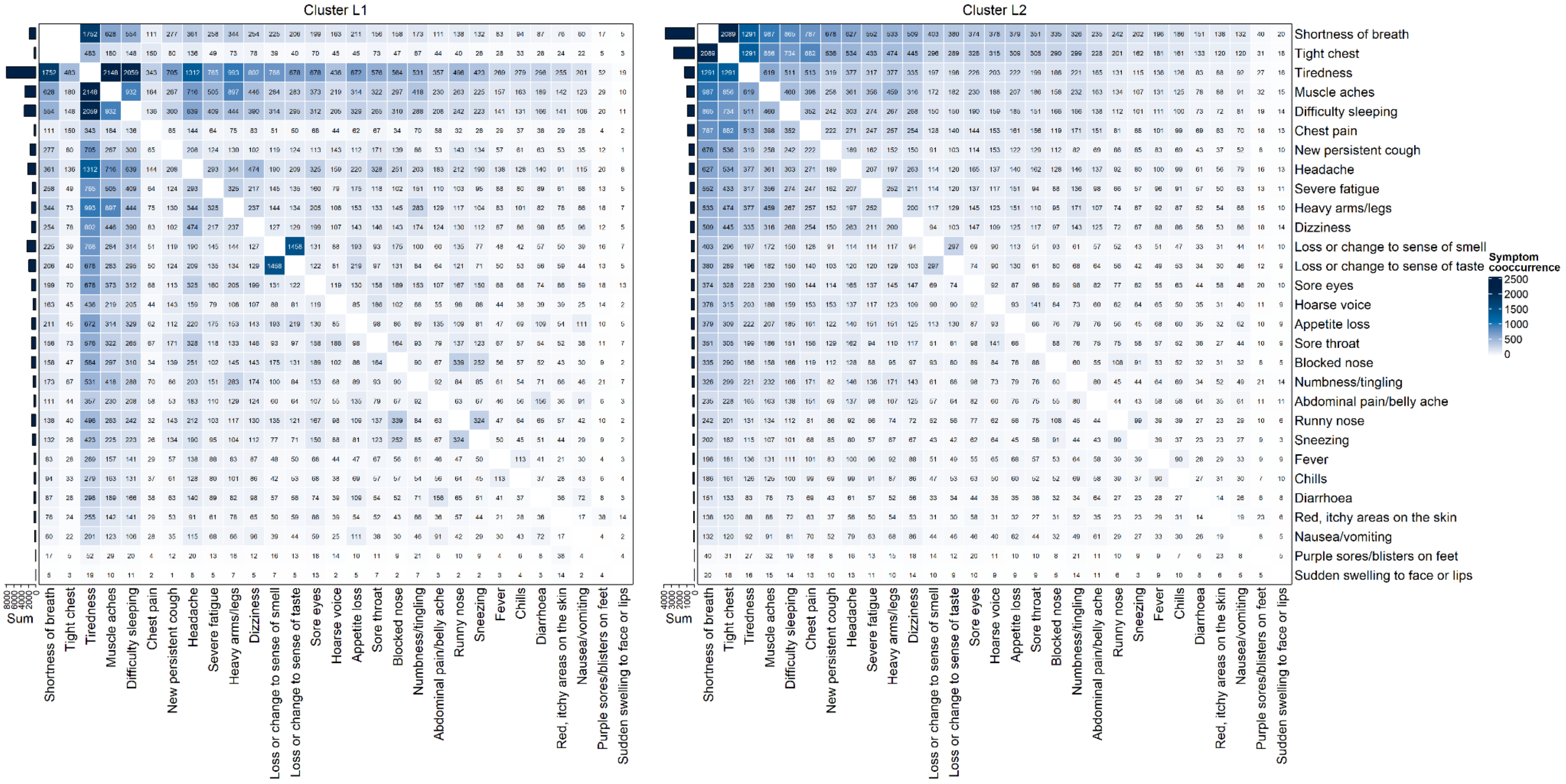

Figure S5 Visualisation of cluster separation and symptom contribution, using MCA.

Panel plot visualising cluster membership using multiple correspondence analysis (MCA) to project categorical symptom data into new continuous components (dimensions). Bar plots show the contribution of the top 10 variables (symptoms) to the displayed dimensions. The medoids (representative observations that form the cluster centres) are marked in the scatter plot.

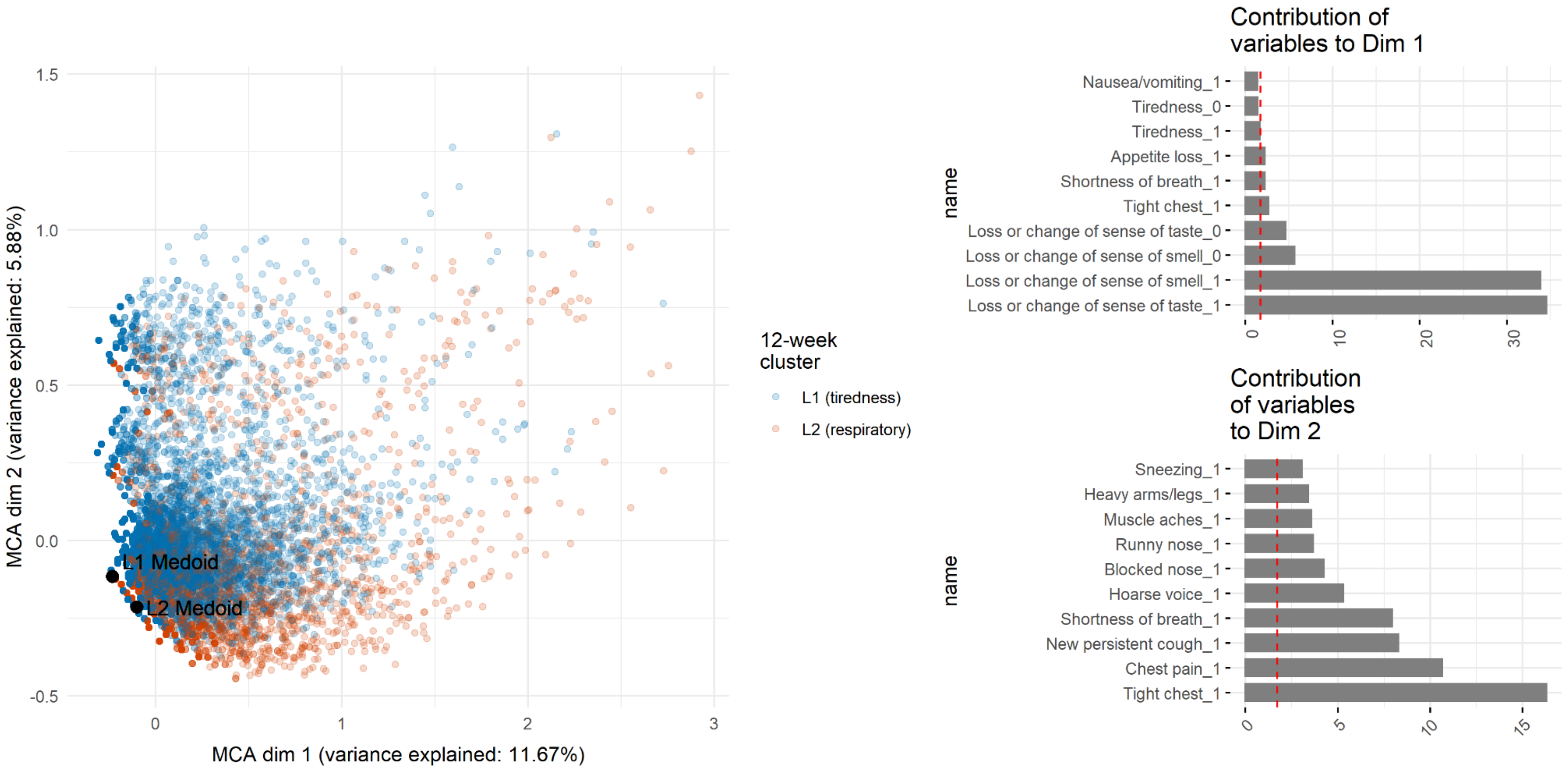

Figure S6 Clustering results (round 6 replication).

Results of clustering on symptom profile at 12 weeks in (A) rounds 3–5, and (B) round 6, using 27 symptoms (as 23 symptom groups) that were common to rounds 3–5 and round 6. CLARA (partitioning around medoids) algorithm was used with the number of clusters constrained to two. Asterisks indicate symptoms that were grouped in round 6. Chills and heavy arms/legs were not surveyed in round 6. In both populations, the clustering identifies one cluster characterised by shortness of breath, accompanied by higher prevalence of tight/heavy chest/ chest pain.

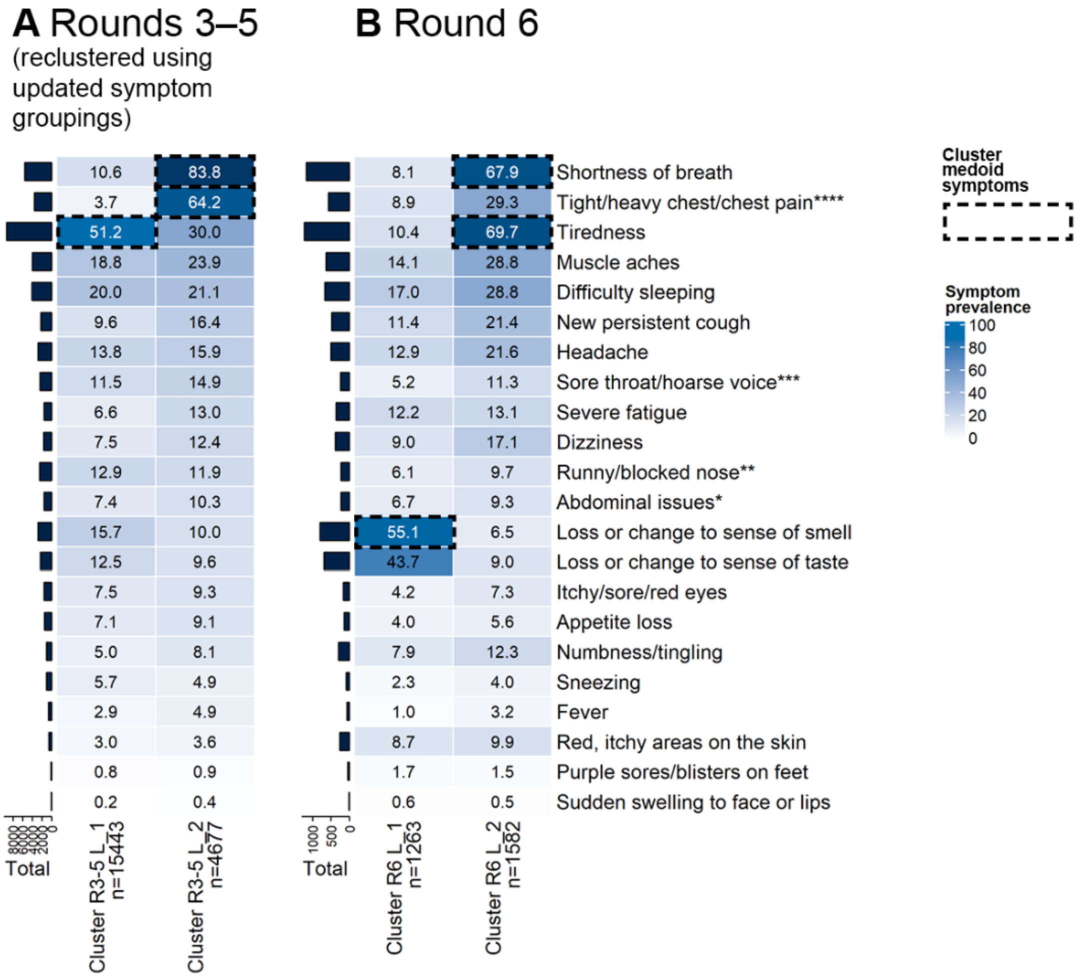

Figure S7 Logistic modelling of PCR-positivity as a function of symptoms experienced.
Results of logistic model with self-reported PCR positivity as the binary outcome variable, and the 29 surveyed symptoms as binary independent variables, among a 30% variable selection holdout data set (n=22,847 observations). The 15 variables that were identified as positively predictive of PCR positivity (shown in bold colours) were taken forward as a sensitivity analysis of prevalence of persistent symptoms beyond 12 weeks, and as outcome variables in logistic regression of risk factors for persistence, among this reduced symptom set. Error bars indicate 95% confidence intervals based on the standard error of the regression coefficient.

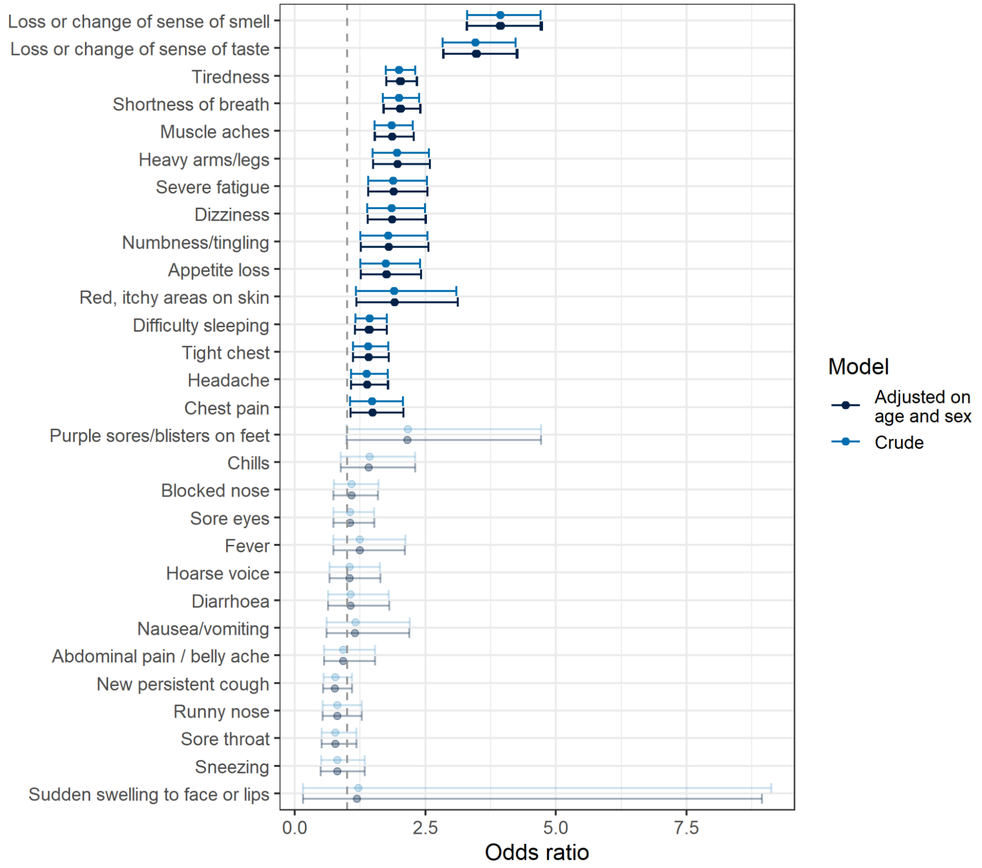

Figure S8 Modelling of persistent symptoms as a function of biological and demographic variables (using 15-symptom subset).

Plots showing the results of predictive modelling, with one or more of a subset of 15 symptoms at 12 weeks (y/n) as the binary outcome variable (n=53,508 observations). Left panel shows odds ratios from logistic regression models, both age-sex and mutually adjusted. Error bars indicate 95% confidence intervals based on the standard error of the regression coefficient. Right panel shows the mean contribution in area under the curve (AUC) that each variable makes to a multivariable boosted tree model, derived using variable permutation. Age, sex, adiposity, current smoker status and gross household income are the strongest predictors of persistent symptoms in multivariable modelling, while Asian ethnicity is associated with a lower risk of persistent symptoms at 12 weeks. Box plots in panel B show median, first and third quartiles; whiskers indicate 1.5 * the interquartile range; data beyond this range are plotted as points.

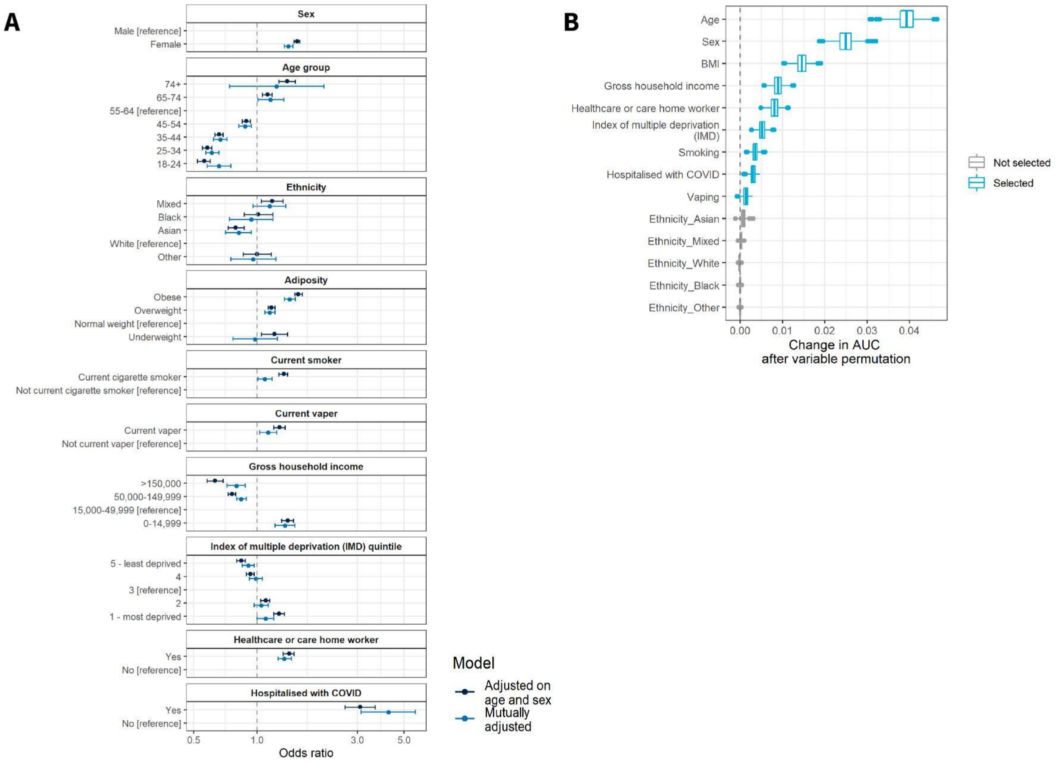

Figure S9 Clustering results (with differing distance metrics).

Results of clustering on symptom profile at 12 weeks in rounds 3–5, using the same algorithm and approach as in the main analysis but replacing Hamming distance with (A) Dice distance and (B) Jaccard distance. Plot (C) shows the average silhouette width across clusters at a range of numbers of clusters using Dice, Jaccard and Hamming distance. Plot (D) shows the cluster membership overlap between the sensitivity analyses (Dice/Jaccard) and the main analysis (Hamming). Two-clusters with Hamming distance (presented in main analysis) created the best clusters according to the silhouette width metric; Dice and Jaccard distance with 5 and 6 clusters respectively each identified two clusters with shortness of breath and other respiratory symptoms (A2 and A5 in both), which in each case included nearly all the observations from the 'respiratory' cluster L2 from the main analysis.

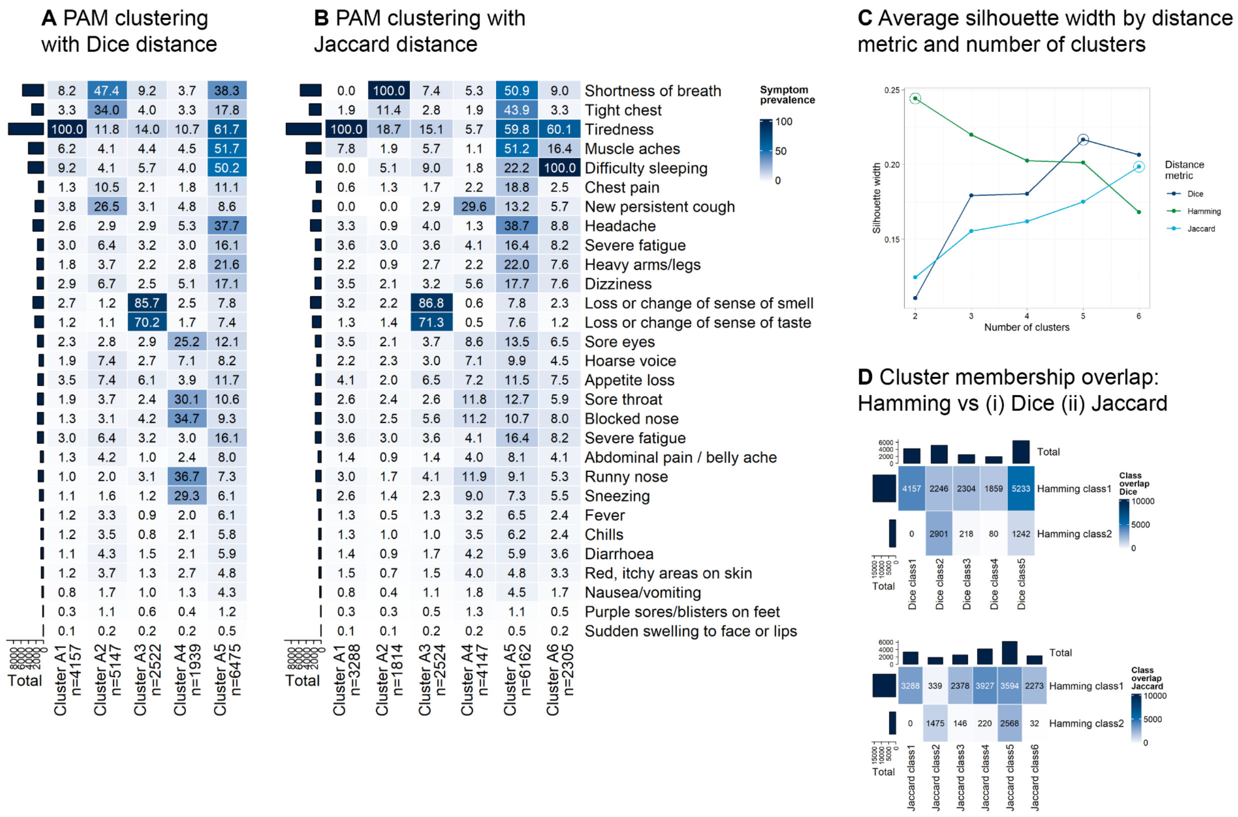

Figure S10 Latent Class Analysis results.
Results of Latent Class Analysis (LCA) on symptom profile at 12 weeks in rounds 3–5, showing (A) symptom prevalence across classes in the two-class model, and (B) average posterior probability across a range of models with different numbers of classes. The two-class model is shown to be optimal. Similar to the main clustering analysis, LCA identifies one larger group in which tiredness is the predominant symptom and one smaller group with multiple symptoms including high prevalence of respiratory symptoms.

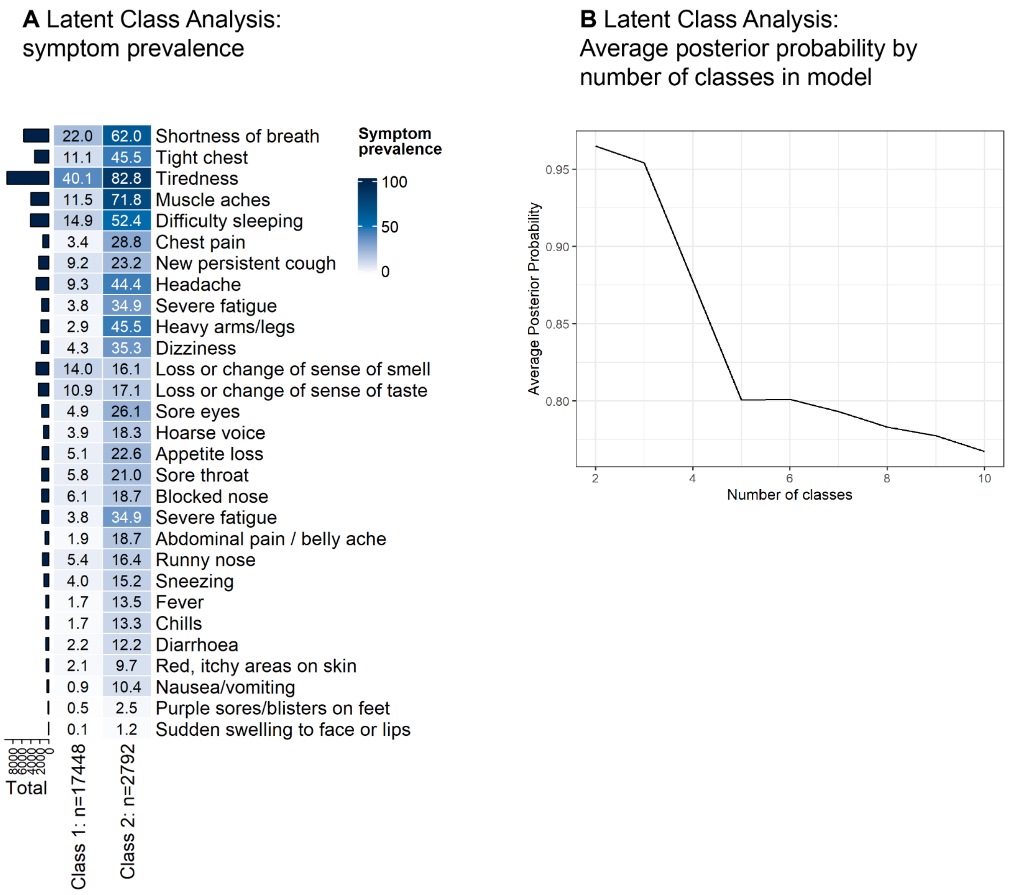

Figure S11 Prevalence of symptoms persisting for 11 or more days in 1,879,842 PCR-negative respondents to REACT-1. Data collected between July 2020 and September 2021 (rounds 2–14). Average weighted prevalence of 11+ days persistent symptoms was 3.06% (3.04,3.09) across rounds 2–14 of REACT-1. Error bars indicate 95% binomial confidence intervals of the prevalence.

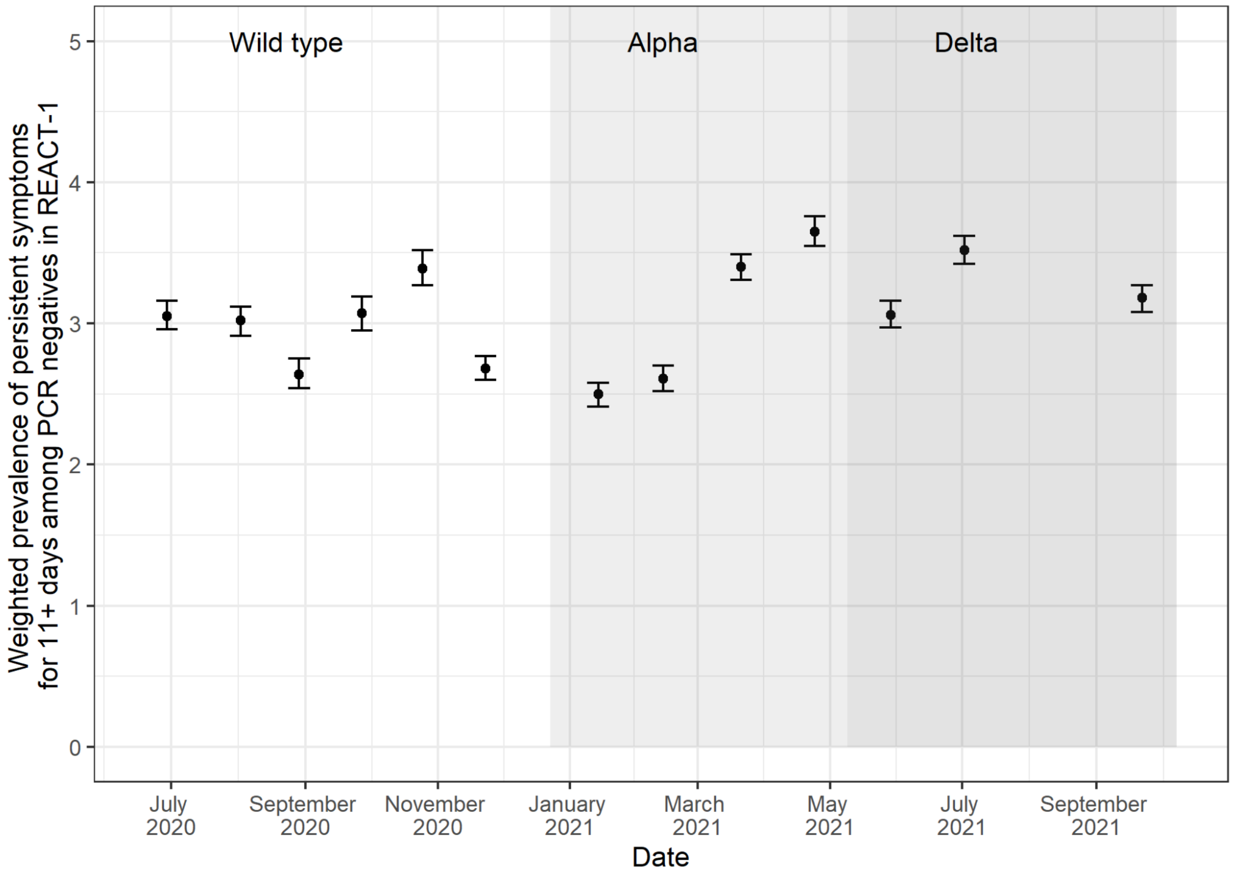

Figure S12 Analysis of the free text responses to COVID-19 symptom questions in REACT-2.
A total of 8,374 respondents left free-text responses, of whom 1,860 reported the described symptoms persisting for 12 weeks or more. From left to right, the panels show (i) analysis of symptoms occurring at time of symptom onset; (ii) analysis of symptoms occurring at 4 weeks after symptom onset, and (iii) analysis of symptoms occurring at 12 weeks after symptom onset. The top two rows of panels visualise within-response word co-occurrence, as networks (top panels) and simple bar plots of the top 20 most frequently co-occurring pairs of words (middle panels). The bottom row shows the most commonly occurring single words.

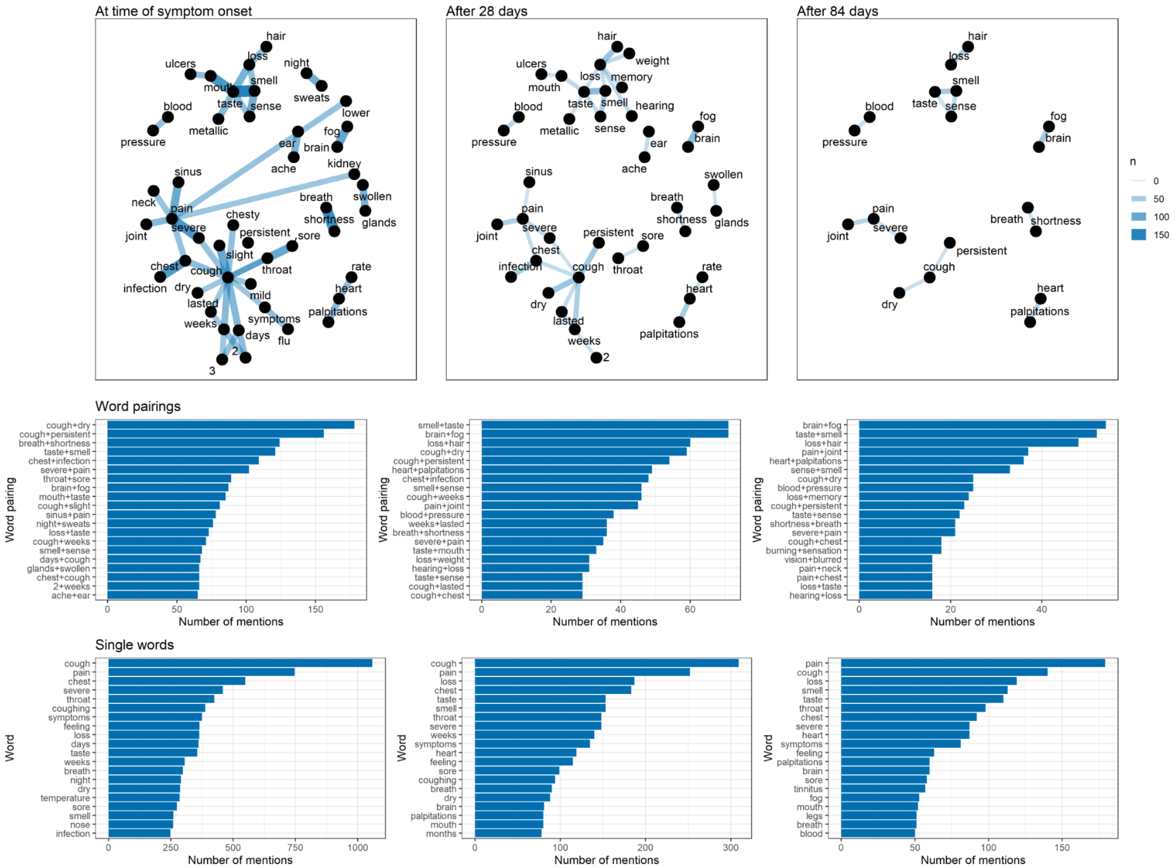

Figure S13 Results of least absolute shrinkage and selection operator (LASSO) stability selection applied to predictors of persistent symptoms at 12 weeks among suspected or confirmed COVID-19.

1000x LASSO models were constructed on 50% subsets of training data; the proportion of models in which each variable was selected represents a measure of variable importance, with a threshold for selection chosen using an internal calibration metric. Top panel shows averaged odds ratios for each predictor across all models in which the predictor was selected; middle panel shows the proportion of models in which each variable was selected; bottom panel shows the area under the curve (AUC) on unseen data of models constructed adding each variable in order of selection proportion, with weightings derived from the average odds ratio. Selected variables were consistent with the results from the main multivariable analysis: age, sex, adiposity, gross household income, hospitalisation with COVID-19, Index of Multiple Deprivation (IMD) and healthcare worker status were selected as predictors of persistent symptoms, conditional on symptomatic infection. Error bars on the top panel indicate the 95% confidence interval of the standard deviation of the distribution of log odds ratios. On the bottom panel error bars indicate 95% bootstrap confidence intervals based on 1,000x resamples.

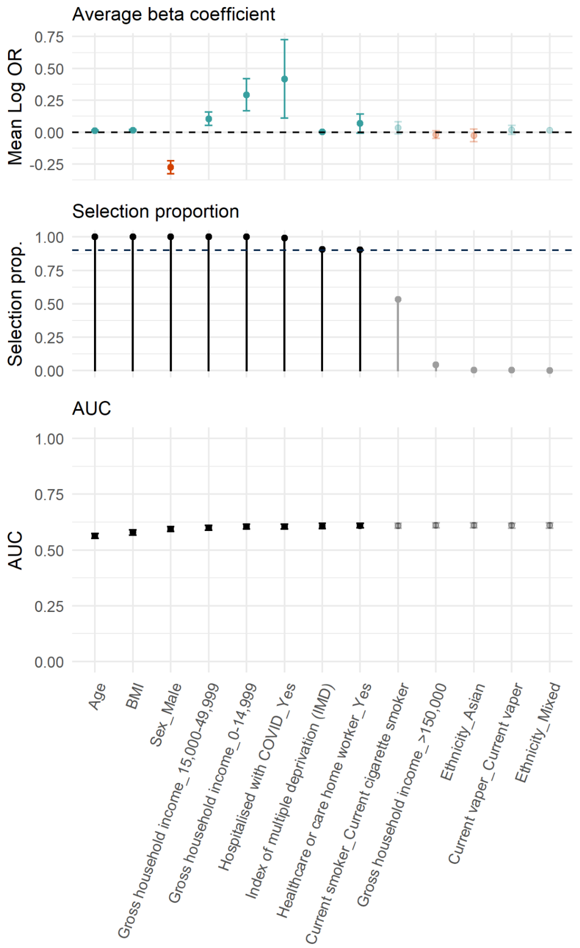

Figure S14 Distribution of dates of self-reported COVID-19 illness.
Plot shows the main study cohort (REACT-2 rounds 3–5, surveyed between October 2020 and February 2021) in dark blue and the validation cohort (REACT-2 round 6, surveyed in May 2021) in light blue; 82% of illnesses in the main study cohort, and 61% in the validation cohort, were 'first wave' infections, i.e. occurring before 1 July 2020.

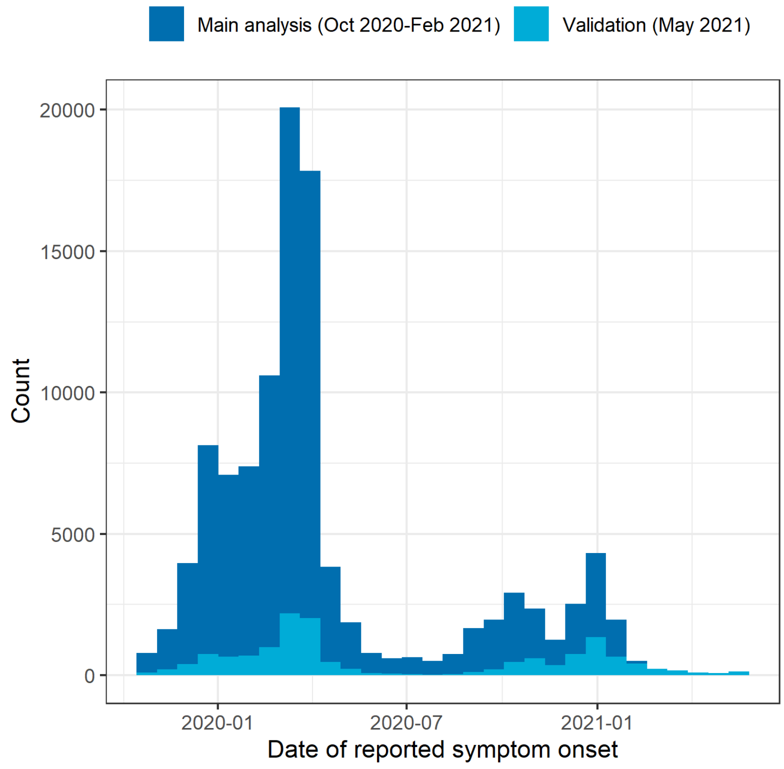

Supplementary methods

Modelling approaches

We took three broad modelling approaches:

1. Logistic regression models, to identify associations between putative risk factors and persistent symptoms at 12 weeks.
2. Variable ranking and selection using Gradient Boosting Models, to identify and rank a parsimonious set of predictors of persistent symptoms at 12 weeks.
3. Generalised Additive Modelling, to explicitly model the relationship between age and risk of persistent symptoms. Specifically, we wanted to see if the relationship was linear across all ages and if the risk gap between men and women persisted at all ages.

1. Logistic regression

To identify associations between putative risk factors and persistent symptoms at 12 weeks, we constructed logistic regression models. The models were specified as follows:

Age-sex adjusted model:

$$y\ [symptoms\ at\ 12\ weeks\ y/n] \sim\ \alpha\ +\ \beta_1[risk\ factor]\ + \beta_2\ [age]\ + \beta_3[sex]$$

Mutually adjusted model:

$$y\ [symptoms\ at\ 12\ weeks\ y/n] \sim\ \alpha\ +\ \beta_1[risk\ factor]\ + \beta X[matrix\ of\ confounders]$$

Age-sex adjusted and mutually adjusted odds ratios and 95% confidence intervals were reported.

2. Variable ranking and selection

For variable selection, we used a multivariable gradient boosting model combined with variable permutation analysis. To capture possible non-linearities and interaction effects, a gradient boosted tree model was used. Catboost has been shown to outperform XGBoost and LightGBM in terms of accuracy and computational expense (https://catboost.ai/#benchmark) and so was selected for this analysis.

This variable selection approach was used in two analyses: 1) to identify a sparse set of predictors of persistent symptoms beyond 12 weeks (i.e. with any symptom y/n as the outcome and a set of biological and sociodemographic variables as predictors), and 2) to identify a sparse set of symptoms that were predictive of a positive LFIA test, for the sensitivity analysis.

First, a model was trained on 80% of the data. Overfitting was controlled by further splitting these data into a training pool and a validation pool. The model was fitted on the training pool, and after each round (each additional tree) performance was evaluated on the validation pool. Model training ceased once performance stopped improving on the validation pool. The objective function was logloss. Default settings were used for all other model hyperparameters.

Next, the model was used to predict the outcome variable in the 20% holdout data, and the AUC recorded.

Next, we permuted each variable in turn in the holdout data and ran the predictions again, recording the AUC. This process was repeated 1000x to obtain a distribution of AUCs for the model with each of the variables permuted. The proportion of permuted runs where the AUC was greater than the unpermuted prediction run was taken as a p-value that the variable was contributing to predictive accuracy. Variables whose proportion was below 5% ($p<0.05$) were selected and highlighted in Figure 2B.

As a complement to this multivariable modelling approach we used logistic least absolute shrinkage and selection operator (LASSO) models with stability selection. Briefly, 1000x LASSO models were constructed on 50% subsets of training data; the proportion of models in which each variable was selected represents a measure of variable importance, with a threshold for selection chosen using an internal calibration metric.

3. *Generalised additive models*

To explicitly model the relationship between age and risk of persistent symptoms we constructed generalised additive models with persistent symptoms at 12 weeks as the binary outcome variable. The model specification was:

$$g(y\ [symptoms\ at\ 12\ weeks\ y/n]) \sim \alpha + f_1[age, sex] + \beta_2\ [sex]$$

… where g is a link function and f_1 represents a smooth function (thin plate spline).

Clustering: detailed methodology

Clustering was performed using the Clustering Large Applications (CLARA) extension to the Partitioning around Medoids (PAM) clustering algorithm. CLARA uses a sampling approach – taking a proportion of observations in a data set and applying the PAM algorithm to select the optimal medoids by minimising the distance between the medoids and cluster members. The clusters are then generalised to the full data set by assigning all observations to the nearest

medoid. We used a 50% data sample as the largest sample size that remained computationally feasible for the main analysis and bootstrap stability analysis.

In the main analysis, we used Hamming distance to quantify distance between observations, preferring it to e.g. the Jaccard/Dice metrics as it quantifies both positive and negative agreement in the distance denominator (while Jaccard and Dice only quantify positive agreement between vectors – i.e. where both participants have a symptom) and in this study we wanted to group respondents on the basis of both presence and absence of symptoms. The CLARA/PAM algorithm was used as it handles binary data and is computationally feasible, with findings and methods that are interpretable to non-experts. The identification of medoids as 'typical' observations, which PAM does, was also desirable as it aids interpretation of the clusters.

As a sensitivity analysis, we re-ran the clustering using the Dice and Jaccard distance metrics to examine how the clusters differ when the distance metric counts only positive agreement (i.e. when two people both have a symptom) in the numerator of the formula.
Where:
a = {1,1} (both respondents have a symptom)
b = {0,0} (neither respondents have a symptom)
c = {1,0} (first respondent has symptom, second does not)
d = {0,1} (second respondent has symptom, first does not)

Hamming distance
(a+b) / (a+b+c+d)

Dice distance
2a / (2a+c+d)

Jaccard distance
a / (a+c+d)

Latent Class Analysis (LCA)

As a further sensitivity analysis, and to test the generalisibility of the structure identified in clustering, we applied Latent Class Analysis to the symptom data at 12 weeks. Observations were assigned to the class with the highest posterior probability, and the classes were characterised in the same way as the clusters (heatmaps showing symptom prevalence by class).

Replication analysis

We repeated our main analyses in data from round 6 of REACT-2, which was gathered between 12 and 25 May 2021. The question structure in round 6 was different to rounds 3–5 (see survey questions below) and there were some differences in the symptoms surveyed (see Table S13).

Table S13 Details of symptoms surveyed in rounds 3–5 and in round 6 of REACT-2. X indicates that the symptom was surveyed. Symptoms that were grouped in round 6 are indicated by XX.

Symptom	Surveyed in rounds 3–5	Surveyed in round 6
Chills	X	
Heavy arms/legs	X	
Nausea/vomiting	X	XX (Abdominal issues)
Diarrhoea	X	XX (Abdominal issues)
Abdominal pain/belly ache	X	XX (Abdominal issues)
Runny nose	X	XX (Runny/blocked nose)
Blocked nose	X	XX (Runny/blocked nose)
Sore throat	X	XX (Sore throat/hoarse voice)
Hoarse voice	X	XX (Sore throat/hoarse voice)
Tight chest	X	XX (Tight/heavy chest/chest pain)
Chest pain	X	XX (Tight/heavy chest/chest pain)
Appetite loss	X	X
Sneezing	X	X
Sore eyes	X	X
Loss or change to sense of smell	X	X
Loss or change to sense of taste	X	X
Headache	X	X
Dizziness	X	X
Shortness of breath	X	X
New persistent cough	X	X
Fever	X	X
Difficulty sleeping	X	X
Tiredness	X	X
Severe fatigue	X	X
Numbness/tingling	X	X
Muscle aches	X	X
Red, itchy areas on the skin	X	X
Sudden swelling to face or lips	X	X
Purple sores/blisters on feet	X	X
Thrombosis		X
Confusion/brain fog/forgetfulness		X
Heart issues		X

Pain in joints	X
Weight loss	X
Vision issues	X
Hearing issues	X
Hair loss	X

Survey (Rounds 3–5)

Survey questionnaires for all rounds of REACT-2 can be accessed here. The key questions for self-reported COVID-19, symptoms and symptom duration in rounds 3–5 were:

Before you took this antibody test, did you think you had had COVID-19?

1. Yes, confirmed by a positive test (swab/*PCR/antigen test) (A swab/PCR/antigen test is done by a nasal or throat swab and tests for current COVID-19 infection)
2. Yes, suspected by a doctor but not tested
3. Yes, my own suspicions
4. No

How severe was your illness when you had/thought you had COVID-19?

1. 1. No symptoms
2. 2. Mild symptoms – didn't affect my daily life
3. 3. Moderate symptoms – some effect on my daily life
4. 4. Severe symptoms – significant effect on my daily life

When did your first symptoms start (as best as you can remember)?
[WRITE IN DATE DAY/MONTH/YEAR]

Which of the following symptoms were part of your COVID-19 illness?
[LIST OF 29 SYMPTOMS WITH CHECKBOXES]

Thinking about [EACH SYMPTOM SELECTED ABOVE] do you still have this symptom:

1. Yes
2. No

Thinking about [EACH SYMPTOM SELECTED ABOVE], how long did this symptom last for approximately? Please enter a value for either Days or Weeks. If you are unsure, please give an estimate.
[WRITE IN DURATION]

How severe was your illness when you had/thought you had COVID-19?

1. No symptoms
2. Mild symptoms – didn't affect my daily life
3. Moderate symptoms – some effect on my daily life
4. Severe symptoms – significant effect on my daily life

Survey (Round 6)

The key questions for self-reported COVID-19, symptoms and symptom duration in rounds 3–5 were:

Before you took this antibody test, did you think you had had COVID-19?

5. Yes, confirmed by a positive test (swab/*PCR/antigen test) (A swab/PCR/antigen test is done by a nasal or throat swab and tests for current COVID-19 infection)
6. Yes, suspected by a doctor but not tested
7. Yes, my own suspicions
8. No

How severe was your illness when you had/thought you had COVID-19?

5. 1. No symptoms
6. 2. Mild symptoms – didn't affect my daily life
7. 3. Moderate symptoms – some effect on my daily life
8. 4. Severe symptoms – significant effect on my daily life

When did your first symptoms start (as best as you can remember)?
[WRITE IN DATE DAY/MONTH/YEAR]

Which of the following symptoms were part of your COVID-19 illness?
[LIST OF 29 SYMPTOMS WITH CHECKBOXES]

Some people who have COVID-19 have symptoms that last for more than four weeks. When you had/thought you had COVID-19, did your symptoms last for more than four weeks?

1. Yes, and I still have symptoms
2. Yes, but I no longer have symptoms
3. No, the symptoms lasted for four weeks or less
4. No, never had any symptoms

5. Don’t know – still have symptoms but not yet for four weeks

Thinking of the symptoms that lasted for more than four weeks, which, if any, of the following did you have? Please select all the symptoms you had for more than four weeks, whether or not you saw a doctor.
[LIST OF 30 SYMPTOMS WITH CHECKBOXES]

Thinking about [EACH SYMPTOM SELECTED ABOVE], how long did this symptom last for approximately? If you still have this symptom, how long has it lasted for? If you are unsure, please give an estimate.

1. Less than four weeks
2. Four weeks up to two months
3. Two months up to three months
4. Three months up to six months
5. More than six months
