# Supplementary material for: Persistent COVID-19 symptoms in a community study of 606,434 people in England
Source: Nat Commun. 2022 Apr 12;13:1957. doi: 10.1038/s41467-022-29521-z (PMC9005552; doi:10.1038/s41467-022-29521-z)
Supplement: Supplementary file 2 — Reporting Summary [file 41467_2022_29521_MOESM2_ESM.pdf]

nature portfolio

Corresponding author(s): Prof Paul Elliott

Last updated by author(s): 28-02-2022

Reporting Summary

Nature Portfolio wishes to improve the reproducibility of the work that we publish. This form provides structure for consistency and transparency in reporting. For further information on Nature Portfolio policies, see our Editorial Policies and the Editorial Policy Checklist.

Statistics

For all statistical analyses, confirm that the following items are present in the figure legend, table legend, main text, or Methods section.

n/a	Confirmed	
☐	☒	The exact sample size (*n*) for each experimental group/condition, given as a discrete number and unit of measurement
☐	☒	A statement on whether measurements were taken from distinct samples or whether the same sample was measured repeatedly
☐	☒	The statistical test(s) used AND whether they are one- or two-sided<br>*Only common tests should be described solely by name; describe more complex techniques in the Methods section.*
☐	☒	A description of all covariates tested
☒	☐	A description of any assumptions or corrections, such as tests of normality and adjustment for multiple comparisons
☐	☒	A full description of the statistical parameters including central tendency (e.g. means) or other basic estimates (e.g. regression coefficient) AND variation (e.g. standard deviation) or associated estimates of uncertainty (e.g. confidence intervals)
☐	☒	For null hypothesis testing, the test statistic (e.g. *F*, *t*, *r*) with confidence intervals, effect sizes, degrees of freedom and *P* value noted<br>*Give P values as exact values whenever suitable.*
☒	☐	For Bayesian analysis, information on the choice of priors and Markov chain Monte Carlo settings
☒	☐	For hierarchical and complex designs, identification of the appropriate level for tests and full reporting of outcomes
☒	☐	Estimates of effect sizes (e.g. Cohen's *d*, Pearson's *r*), indicating how they were calculated

Our web collection on statistics for biologists contains articles on many of the points above.

Software and code

Policy information about availability of computer code

Data collection	All data collection for the REACT2 study is captured with Questback (Spring 2020 installation).
Data analysis	All data were analysed using R v4.0.5 (2021). The Partitioning Around Mediods Algorithm was used for clustering, from the 'fpc' R package, version 2.2-9 (2020). Latent Class Analysis was conducted with the poLCA R package, version 1.4.1 (2016). Gradient boosted tree modelling was conducted with Catboost, version 1.0.1, (2021).

For manuscripts utilizing custom algorithms or software that are central to the research but not yet described in published literature, software must be made available to editors and reviewers. We strongly encourage code deposition in a community repository (e.g. GitHub). See the Nature Portfolio guidelines for submitting code & software for further information.

Data

Policy information about availability of data

All manuscripts must include a data availability statement. This statement should provide the following information, where applicable:

- Accession codes, unique identifiers, or web links for publicly available datasets
- A description of any restrictions on data availability
- For clinical datasets or third party data, please ensure that the statement adheres to our policy

The original datasets generated or analysed, or both, during this study are not publicly available because of governance restrictions and the identifiable nature of the data. Summary data is available here: https://github.com/mrc-ide/reactidd/tree/master/inst/extdata/react2_long_COVID_paper

Field-specific reporting

Please select the one below that is the best fit for your research. If you are not sure, read the appropriate sections before making your selection.

☒ Life sciences ☐ Behavioural & social sciences ☐ Ecological, evolutionary & environmental sciences

For a reference copy of the document with all sections, see nature.com/documents/nr-reporting-summary-flat.pdf

Life sciences study design

All studies must disclose on these points even when the disclosure is negative.

Sample size	Sample size for main analysis was n=508,707, from REACT-2 rounds 3–5. Replication analysis was conducted in n=97,717 from REACT-2 round 6. Total sample size therefore n=606,434.<br><br>The REACT-2 study is powered conservatively to give information on every LTLA in England (n=315), in each round, under the assumption that seroprevalence in each Local Authority is independent. Full information on sample size calculation and rationale is available in the protocol paper: https://wellcomeopenresearch.org/articles/5-200/v2.
Data exclusions	Asymptomatic respondents or those reporting symptom onset <12 weeks prior to response date were excluded (n=432,552), leaving a final study population of 76,155.
Replication	Replication analysis was conducted in n=97,717 from REACT-2 round 6. The main analyses replicated in round 6: similar patters were observed in the clustering analysis and the same risk factors were identified. Overall prevalence estimates were lower in round 6, as reported.
Randomization	Randomisation not applicable in this study design: exposure and disease was self-reported retrospectively.
Blinding	Blinding was not applicable to the study design: the lateral flow test results used in REACT-2 were (i) self-administered and could not be blinded, and (ii) were not used in this analysis. Our exposures and disease were self-reported retrospectively.

Reporting for specific materials, systems and methods

We require information from authors about some types of materials, experimental systems and methods used in many studies. Here, indicate whether each material, system or method listed is relevant to your study. If you are not sure if a list item applies to your research, read the appropriate section before selecting a response.

Materials & experimental systems

n/a	Involved in the study	
☒	☐	Antibodies
☒	☐	Eukaryotic cell lines
☒	☐	Palaeontology and archaeology
☒	☐	Animals and other organisms
☐	☒	Human research participants
☒	☐	Clinical data
☒	☐	Dual use research of concern

Methods

n/a	Involved in the study	
☒	☐	ChIP-seq
☒	☐	Flow cytometry
☒	☐	MRI-based neuroimaging

Human research participants

Policy information about studies involving human research participants

Population characteristics	Full population:<br>n=508,707 (men = 223,861 women=284,840)<br>Mean age = 51.2 years<br><br>Main study population:<br>n=76,155 (men = 32,500; women=43,654)<br>Mean age = 47.2 years<br><br>Replication population:<br>n=97,717 (men = 40,863; women = 56,871)<br>Mean age = 52.4
Recruitment	Random population samples of adults in England were invited to take part every 2–4 months using the National Health Service patient list to achieve similar numbers of participants in each of 315 lower-tier local authority areas. Participants

registered via an online portal or by telephone. Those registered were sent a test kit by post that included a self-administered point-of-care lateral flow immunoassay test with instructions and a link to an online video. Participants also completed an on-line (or telephone) questionnaire where they gave details of whether or not they had had COVID-19, symptoms they had experienced that they related to COVID-19, and duration of those symptoms.

There is the possibility of self-selection/participation bias as the REACT-2 study included a self-administered LFIA: it is plausible that people with persistent symptoms may have been more likely to participate in order to ascertain their antibody status. We have acknowledged this in the limitations section of the discussion.

Ethics oversight	South Central-Berkshire B Research Ethics Committee (IRAS ID: 283787)

Note that full information on the approval of the study protocol must also be provided in the manuscript.
